# Asymmetric Synthesis and Applications of Chiral Organoselenium Compounds: A Review

**DOI:** 10.3390/molecules29153685

**Published:** 2024-08-03

**Authors:** Yanyu Jian, Thishana Singh, Pher G. Andersson, Taigang Zhou

**Affiliations:** 1 College of Chemistry and Chemical Engineering, & Institute for Carbon Neutrality, Southwest Petroleum University, Chengdu 610500, China; jyy960810@163.com; 2 School of Chemistry and Physics, University of KwaZulu-Natal, Private Bag X54001, Durban 4000, South Africa; singht1@ukzn.ac.za; 3 Department of Organic Chemistry, Stockholm University, Svante Arrhenius väg 16C, 10691 Stockholm, Sweden; 4 Tianfu Yongxing Laboratory, Chengdu 610213, China

**Keywords:** chiral organoselenium, asymmetric synthesis, application

## Abstract

The synthesis and application of organoselenium compounds have developed rapidly, and chiral organoselenium compounds have become an important intermediate in the field of medicine, materials, organic synthesis. The strategy of developing a green economy is still a challenge in the synthesis of chiral organoselenium compounds with enantioselective properties. This review covers in detail the synthesis of chiral organoselenium compounds from 1979 to 2024 and their application in the fields of asymmetric synthesis and catalysis.

## 1. Introduction

**Figure 1:**
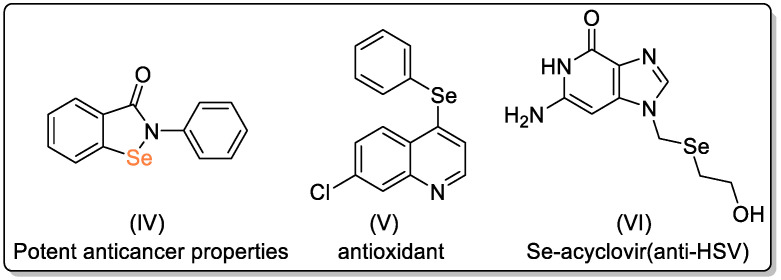
Application of organoselenium compounds.

Selenium is a non-metal element, that was discovered by Swedish chemist Berzelius in 1818 [1]. It is generally found in different inorganic forms in the soil as Se (Se^0^), selenide (Se^2+^), selenate (SeO_4_^2−^), or selenite (SeO_3_^2−^) [2]. Selenium is important to human health and exists in the human body as a trace element in the form of selenocysteine [3]. In recent decades [4], studies have found that organoselenium has antiviral, anti-inflammatory, antitumor, antidepressant, antioxidant, and anticonvulsant activities (Figure 1).

Research in the field of organoselenium began in 1836 with the synthesis of diethyl selenide by Löwig [5,6,7], which was isolated and purified by Rathke 33 years later [8,9]. Because organoselenium compounds are highly malodorous and difficult to purify, the development of organoselenium was slow during this period [10]. During this time only a few simple aliphatic organoselenium compounds were identified such as selenol (RSeH), selenide (RSeR) and diselenide (RSeSeR). From the 1970s, the discovery and identification of different types of organoselenium compounds has increased and has attracted the attention of scientists [11,12,13,14]. Organoselenium compounds has become an emerging field of research. 

Organoselenium compounds are used in supramolecular chemistry. Supramolecular chemistry integrates the four fundamental disciplines of chemistry (organic, inorganic, analytical, and physical) into one. Supramolecular behavior is exhibited through electrostatic action between anions and cations or via hydrogen bonding [15,16,17,18]. Selenium’s unique reactivity ensures regioselectivity and stereoselectivity is achieved. Selenium is often used in organic synthesis as nucleophilic selenophiles, electrophilic selenophilic reagents and free radical selenium reagent [19,20,21,22]. Selenium has a lower bond energy and electronegativity than sulfur, which belongs to the same group in the periodic table. Due to its redox properties, it is easy to remove as well as substitute selenium atoms in a reaction. Hence, current research is mostly focused on the synthesis of non-chiral organic selenium compounds. The main reason is the rapid racemization of intermediates, which hinder the formation of chiral organic selenium compounds. 

**Figure 2:**
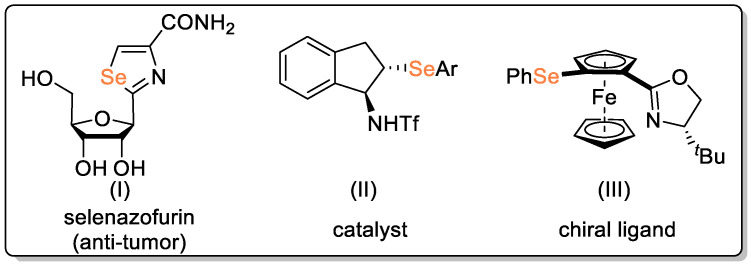
Chiral organoselenium compounds.

In the past decade, the importance of organoselenium compounds, especially chiral organoselenium compounds has been the focus and several groups are committed to developing new strategies for the synthesis of chiral organoselenium compounds. Organoselenium compounds are important building blocks in materials chemistry [23,24,25,26,27]. Chiral organoselenium compounds are indispensable intermediates [28] that can serve as the core backbone of a drug [29,30,31,32]. They also play an important role in organic asymmetric synthesis, for example, chiral catalysts and chiral ligands are used to regulate enantioselectivity, as shown in (Figure 2). Although extensive research has been conducted on various preparation methods and applications of chiral organoselenium compounds, there are few reviews related to the study of chiral organoselenium compounds. As early as 2007, Zhu [33] reported in detail the synthesis strategy of asymmetric organoselenium compounds. However, with the rapid development of asymmetric synthesis in recent years, the synthetic strategy of asymmetric organoselenium compounds has also grown. The review of asymmetric organic selenium compounds needs updating, and the classification should be more comprehensive. Zeng [34] and Back [35] have recently collated literature in this topic, which includes asymmetric processes catalyzed by chiral selenium-based reagents, auxiliaries, and catalysts Selenium-containing catalysts play an important part in numerous reactions. Examples include selenium-ligated palladium (II) complexes as Heck reaction catalysts, organoselenium catalysis in Michael-Type reactions, and organoselenium compounds that catalyze organic asymmetric synthesis. Rodríguez [36] discussed the use of selenium in catalysis and proposed reaction mechanisms. 

**Figure 3:**
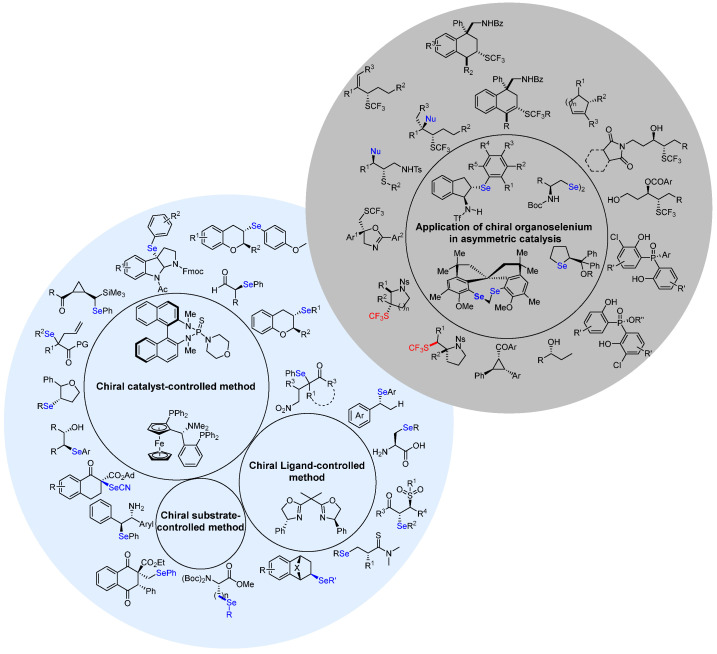
Asymmetric synthesis of organoselenium compounds and its application.

There are three general methods (Figure 3) for the synthesis of chiral organic selenium compounds: (a) chiral substrate-controlled methods; (b) chiral ligand-controlled methods, a chiral auxiliary primarily controls enantioselectivity through complex interactions between chiral ligands and metals [37,38]; (c) chiral catalyst-controlled methods: enantioselectivity is controlled through electronic effects and steric hindrance [39,40,41]. However, no visible boundaries exist, such as asymmetric olefin addition and cyclization, which are frequently aided by chiral ligands or chiral catalysts.

Some chiral auxiliaries, for example, can be utilized as chiral catalysts in asymmetric synthesis as well. Our goal is to summarize progress in the synthesis of enantioselective organoselenium compounds based on their reaction type, which is divided into the following groups: asymmetric cycloaddition, seleniolactonizations, selenioaminations and amides, asymmetric addition reaction, asymmetric selenizing of aldehydes/ketones, asymmetric ring-opening reaction, asymmetric substitution reaction, and asymmetric decarboxylation reaction. The first section of this review covers the synthesis of chiral organic selenium compounds, while the second section discusses the use of chiral organic compounds as catalysts in organic asymmetric synthesis. Enantioselective synthesis of chiral selenium compounds is a rapidly growing topic in modern organic synthesis. Furthermore, chiral selenium compounds have gained considerable attention because to their high potential in catalytic asymmetric synthesis.

Our research group has focused on asymmetric catalytic processes, including asymmetric hydrogenation [42,43,44,45,46], asymmetric radical reaction, and asymmetric C–H activation [47]. Organoselenium compounds have been used in a variety of applications and industries. We began our research on organoselenium compounds in 2020 and published the first example of a selenium-directed C-H borylation procedure for the synthesis of diverse organoselenium compounds [22]. Later in 2022, we reported the first example of radical cyclization and ring-opening of oxime esters using diselenides to synthesize a variety of functionalized organoselenium compounds [21]. To the best of our knowledge, there have been no reports of asymmetric hydrogenation to create chiral organoselenium compounds. Based on our previous research findings, we are focusing on asymmetric hydrogenation and an asymmetric radical strategy for the simple synthesis of organoselenium compounds. So far, we’ve made good progress in the lab. 

## 2. Asymmetric Synthesis of Organoselenium Compounds

Stereoselective processes involving C-Se, Se-Se, Se-O, and Se-N bonds have grown in diversity, efficiency, and applicability [19,48,49,50]. In general, chiral organoselenium compounds are synthesized via three routes. In this section, we describe the asymmetric synthesis of organoselenium compounds using substrate manipulation, chiral additives, and chiral catalysts, that result in a series of optically active organoselenium derivatives.

### 2.1. Chiral Catalyst or Ligand-Controlled Method

#### 2.1.1. Asymmetric Cycloaddition

**Scheme 1:**
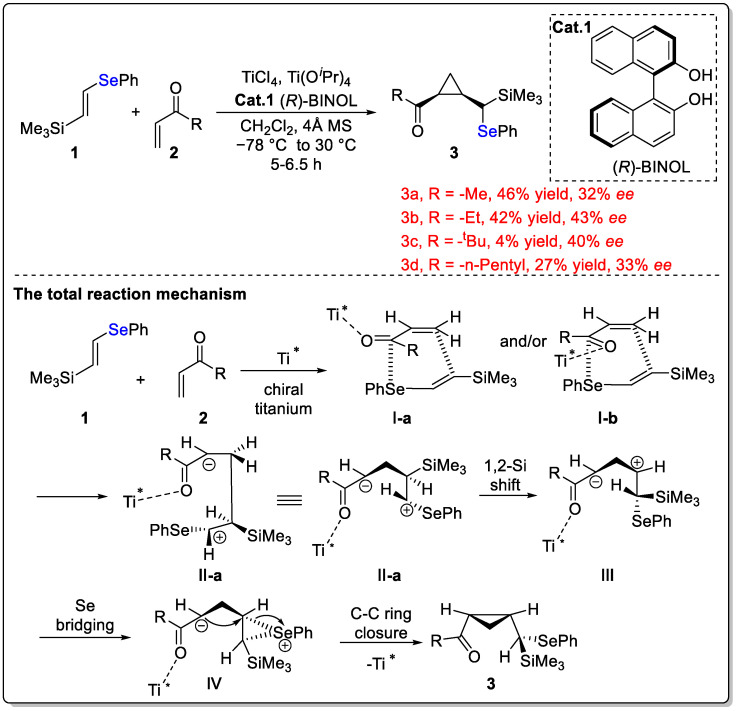
Asymmetric [2 + 1] cycloaddition reaction of 1-phenylselenium-2-silylethylene.

In 1996, Yamazaki and coworkers [51] reported the reaction of (E)-1-(phenylseleno)-2-(trimethylsilyl)ethene **1** and α-unsaturated ketone **2** in the presence of TiCl_4_, Ti(O*i*Pr)_4_, (*R*)-(BINOL) **Cat.1** chiral Lewis acids. The result was a cis-cyclopropane product 3 (Scheme 1) with moderate enantioselectivity (40–57% *ee*) and yields of 4–45%.

Scheme 1 depicts a probable reaction mechanism for the asymmetric [2 + 1] cycloaddition reaction. The first step is to combine vinyl ketone **2** with chiral titanium to generate a chiral titanium-vinyl ketone complex (**I**), which will be attacked by selenosilyl nucleophile **1**. The titanium-vinyl ketone complex (**I**) can take either *s-cis* or *s-trans* configuration. Because of the stable secondary orbital interaction (Se---C=O), synclinal stereoselective addition may have an effect on face selectivity. The succeeding 1,2-silicon migration generates selenium-bridged intermediate (**IV**) by minimum motion and ring closure, yielding cyclopropane **3**.

#### 2.1.2. Selenolactonizations

**Scheme 2:**
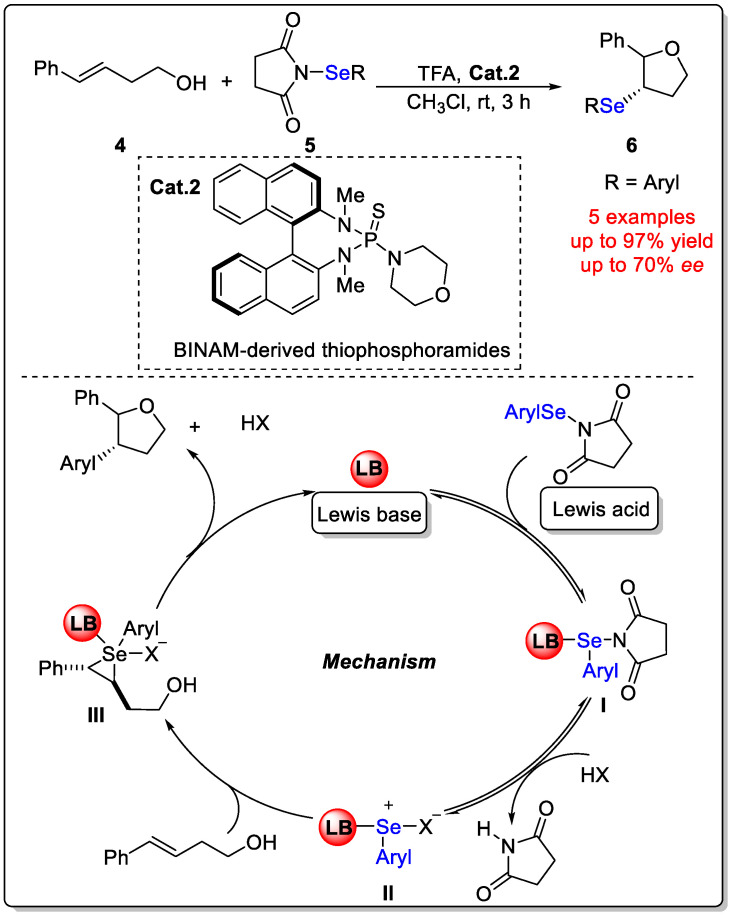
Lewis bases catalyzed asymmetric intramolecular selenium cyclization of unsaturated alcohols.

In 2010, Denmark and coworkers [52] used Lewis base to catalyze the asymmetric intramolecular selenoetherification of unsaturated alcohols **4**. An extensive study of chiral Lewis bases showed that 1,1′-binaphthalene-2,2′-diamine (BINAM)-derived thiophosphoramides catalyzed cyclization of unsaturated alcohols **4** in the presence of N-(2-nitrophenylselenenyl) succinimide **5** and methanesulfonic acid (Scheme 2). A variety of cyclization products **6** were synthesized with good chemical yield (97%) and moderate to good enantioselectivity (70% *ee*). The catalytic and enantioselective selenium functionalization of unactivated olefins were achieved for the first time. The authors investigated the reaction mechanism, revealed that the cycle begins with the reversible binding of Lewis bases to selenimide to create adducts (**I**). Succinimidyl protonation in (**I**) generated the intermediate (**II**). This was followed by the formation of cyclic selenium ions (**III**), which underwent nucleophilic trapping, releasing the product and regenerating the Lewis base catalyst. A mechanism for inhibiting the racemization of selenium ion intermediates (**II**) was discovered. When aryl selenium cation transfer cyclization (**III**) occurs quicker than substrate racemization, high enantioselectivity might be expected.

**Scheme 3:**
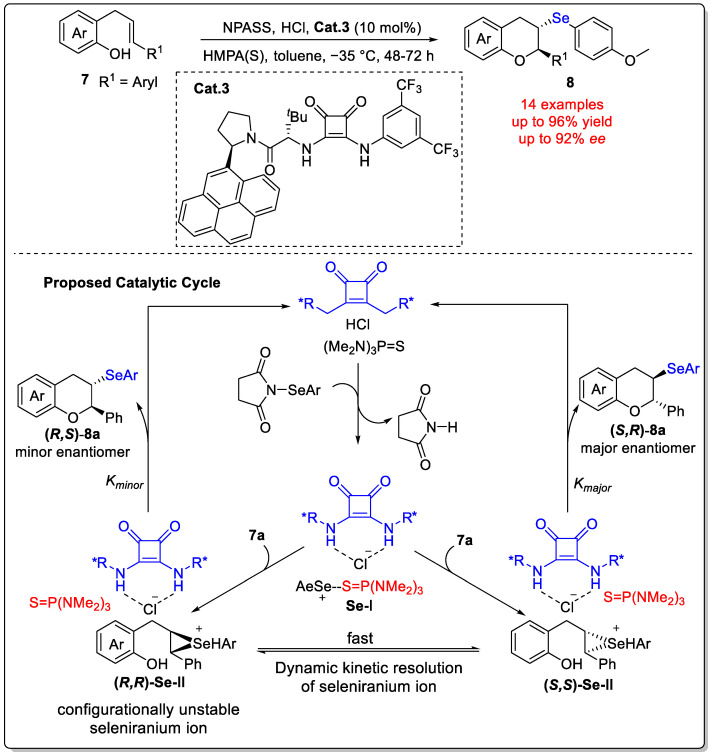
Selenium cyclization catalyzed by chiral square amide catalysts.

In 2014, Jacobsen and coworkers [53] reported a novel chiral squaramide catalyst **Cat.3** in combination with mineral acids and achiral Lewis bases capable of high enantioselective selenocyclization (Scheme 3). The authors selected o-allyl-substituted phenol **7** as the substrate because it can form a chroman-type product with two contiguous stereogenic centers; *N*-*p*-anisylselenyl succinimide (NPASS) as the selenium donor; and hydrogen chloride as a co-catalyst. Activating the electrophilic selenium reagent with Lewis bases and Brønsted acids gave a reactive ion pair that may be linked to the squaramide catalyst. Tris-(dimethylamino)phosphorus sulfide (HMPA(S)) promoted selenocyclization and the products **8** were obtained in excellent enantioselectivity (up to 92% *ee*). The enantioselectivity of recrystallized products increased to 99% *ee*. The strategy had good substrate applicability, and most of the substrates yielded the target products with good to excellent yields and high enantioselectivity. The mechanistic studies revealed that the enantioselectivity of selenium ions was caused by dynamic kinetic resolution via anion-binding catalysis.

**Scheme 4:**
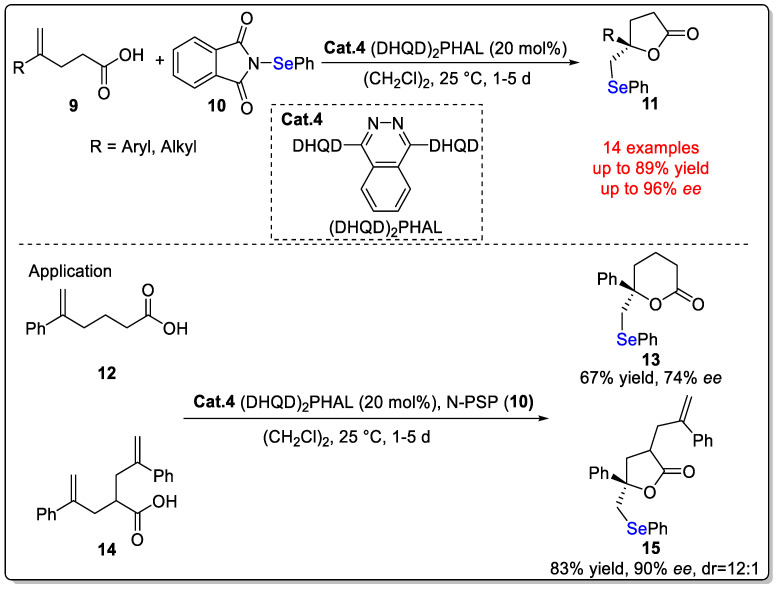
Asymmetric selenolactonization of olefinic acids.

In 2015, Yeung and coworkers [54] reported a catalytic and highly enantioselective selenolactonization of olefinic acids using (DHQD)_2_PHAL **Cat.4** as a bifunctional organocatalyst (Scheme 4). Treatment of olefinic acids **9** and N-phenylselenophthalimide(N-PSP) **10**, used as the electrophilic selenium reagent, gave the corresponding selenolactones **11** with up to 96% *ee*. The substrates scope was extended to include different skeletons **12** under the standard conditions, and gave **13** in 74% *ee*. This catalytic system was applied to diene carboxylic acid **14** where desymmetrization-asymmetric selenolactonization occurred, affording **15** in 90% *ee* with 12:1 dr.

**Scheme 5:**
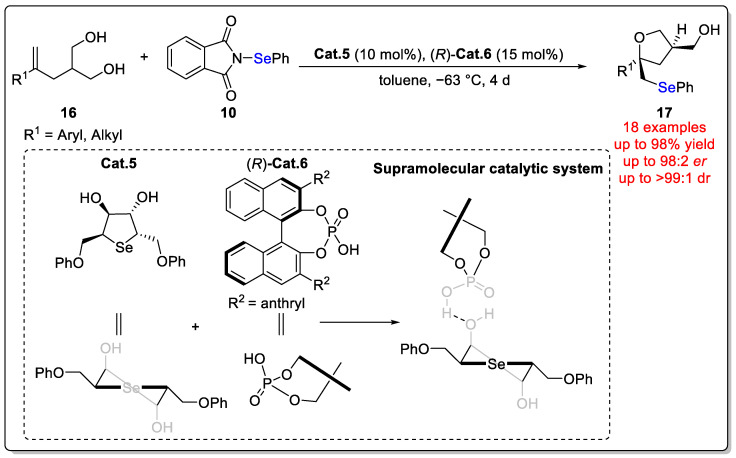
Desymmetrizing enantio- and diastereoselective selenoetherification of olefinic 1,3-diol via supramolecular catalysis.

In 2018, Yeung, Wong and Ke [55] developed asymmetric selenocyclization and desymmetrization of olefinic 1,3-diol, This is promoted by a unique chiral pairing between a C_2_-symmetric cyclic selenide **Cat.5** catalyst as the Lewis base and a chiral BINOL-derived phosphoric acid **Cat.6** as the Brønsted acid cocatalyst (Scheme 5). In this study olefinic 1,3-diol **16** was the substrate and N-phenylselenophthalimide (N-PSP) **10** was the electrophilic selenium reagent. A range of phenylseleno-functionalized tetrahydrofuran **17** were synthesized with good to excellent diastereo- and enantioselectivity (up to 98% yield; up to 98:2 er and >99:1 dr). The tetrahydrofuran products **17** contained a phenylselenoether moiety, that was further transferred to useful synthetic compounds, such as aldehydes. Mechanistic studies and theoretical calculations show that chiral **Cat.5**/(*R*)-**Cat.6** pairs form a supramolecular catalytic system via hydrogen bonds in the selenocyclization reaction.

#### 2.1.3. Selenoaminations and Amidations

**Scheme 6:**
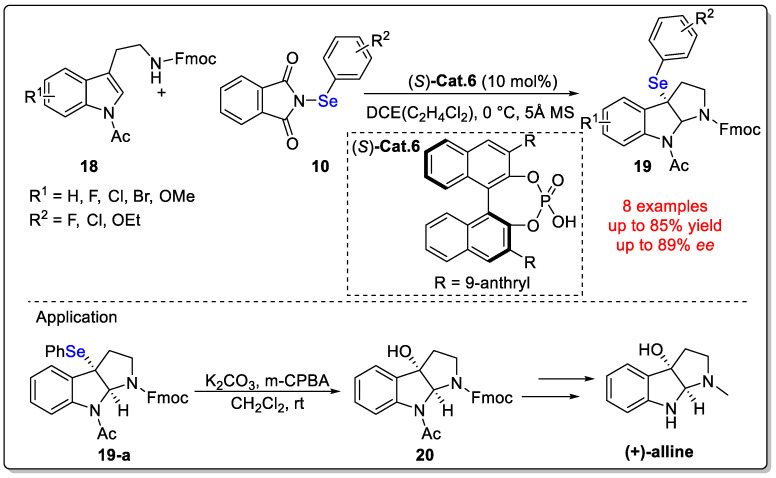
Chiral phosphoric acid catalyzes the synthesis of 3a-(phenylselenyl)-1,2,3,3a,8,8a-hexahydropyrrolo[2,3-b]indole derivatives.

In 2013, Gong and coworkers [56] reported selenofunctionalization of tryptamine derivatives catalyzed by chiral phosphoric acid to afford 3a-(phenylselenyl)-1,2,3,3a,8,8a-hexahydropyrrolo[2,3-b]indole derivatives (Scheme 6). Treatment of tryptamine derivatives **18** and N-(phenylseleno)phthalimide (*N*-PSP) **10** in the presence of chiral phosphoric acid **Cat.6** yielded 3a-(phenylselenyl)-1,2,3,3a,8,8a-hexahydropyrrolo[2,3-b]indole derivatives **19** in good yield (up to 85%) and with good enantioselectivity (up to 89% *ee*). Furthermore under alkaline conditions, the oxidative deselenation of **19-a** with *m*-CPBA produced the corresponding alcohol **20** in 95% yield, which could be subsequently converted to the chiral precursor (+)-alline. The alcohols exhibited the same stereochemistry as the parent selenide.

**Scheme 7:**
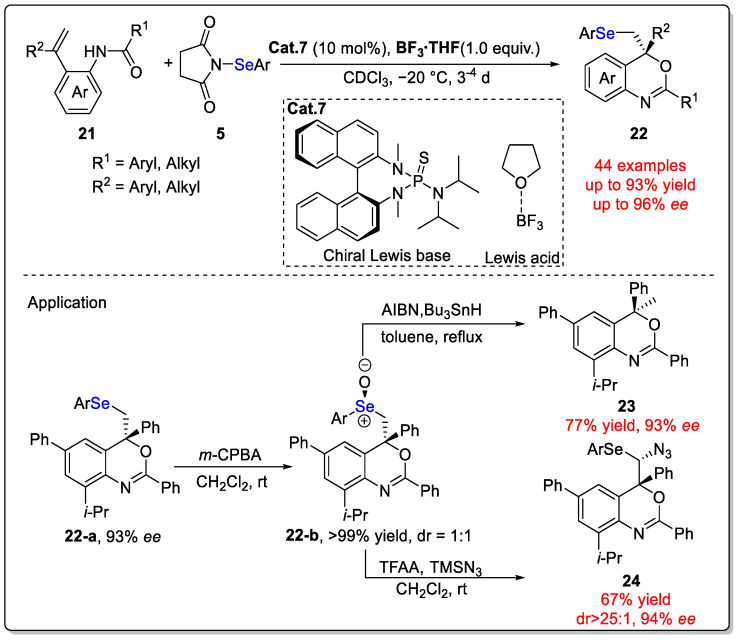
Asymmetric synthesis of selenium-containing 4H-3,1-benzoxazines.

In 2022, Chen and coworkers [57] reported the first enantioselective selenocyclization of 1,1-disubstituted alkenes **21**. They achieved this by combining chiral BINAM-derived sulfides **Cat.7** and achiral Lewis acids BF_3_▪THF to produce chiral oxaquaternary stereocenters (Scheme 7). Various selenium-containing 4H-3,1-benzoxazine derivatives **21** were obtained in moderate to good yields (up to 93%) and good to excellent enantioselectivities (up to 96% *ee*). Product **22-a** was oxidised to selenoxide **22-b** with m-chloroperbenzoic acid with excellent yield (>99%). Product **22-b** generated product **23** (77% yield and 93% *ee*) via reductive deselenenylation using AIBN/Bu_3_SnH. The Seleno−Pummerer reaction with TFAA and TMSN_3_ gave product **24** (67% yield, >25:1 dr, 94% *ee*).

#### 2.1.4. Asymmetric Addition Reaction

**Scheme 8:**
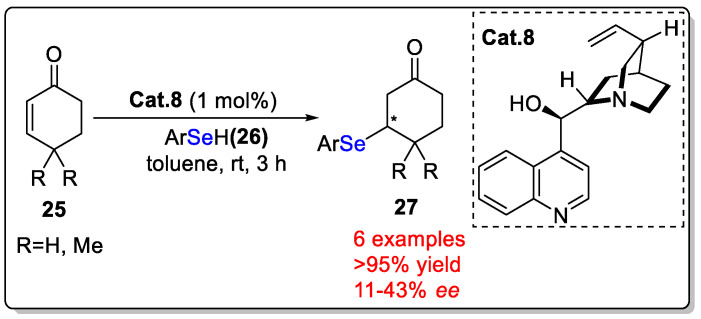
Asymmetric catalytic addition of aryl selenol with 2-cyclohexen-1 one.

In 1979, Wynberg and Pluim [58] reported the first enantioselective synthesis of organoselenium compounds using (−) cinchonidine **Cat.8** (Scheme 8). The asymmetric catalytic addition between aryl selenol **26** and 2-cyclohexen-1-ketone **25** was achieved. The target products **27** were generated in excellent yields (>95%) and enantioselectivity (11–43% *ee*).

**Scheme 9:**
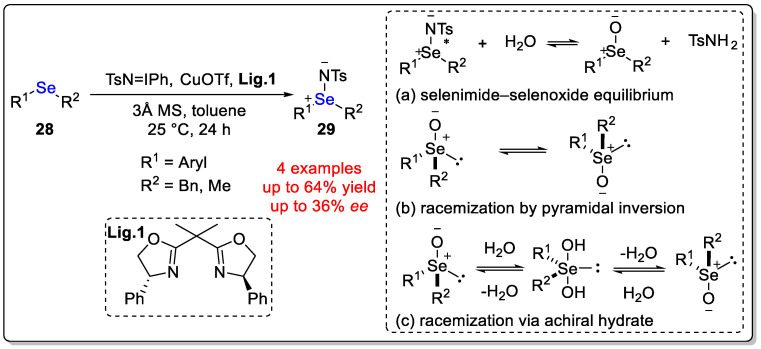
Asymmetric imidation of aryl selenides.

In 2000, Uemura and coworkers [59] reported aryl benzyl selenide **28** reacted with N-(*p*-toluenesulfonyl)imino]phenyliodinane [TSN = IPH] in toluene or acetonitrile to give the corresponding N-*p*-toluene selenimide **29** in yields of 31–64% and 20–36% *ee* (Scheme 9). Enantioselective imidation was used in the presence of chiral bis (oxazoline) ligands **Lig.1** and molecular sieve to produce N-*p*-toluene selenimide, with best being 2-naphthylbenzylselenimide (64% yield, 36% *ee*). The addition of molecular sieves remove water in the reaction. This stops the equilibrium between selenimide and selenium oxide, which is known to racemize rapidly [60,61,62,63]. Hence, removing water is a key step to control the enantioselectivity of the reaction.

**Scheme 10:**
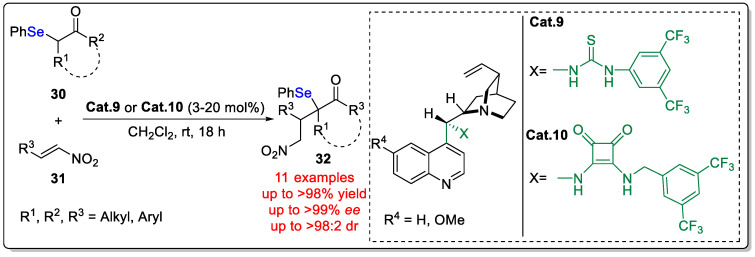
Asymmetric synthesis of α-alkyl α-selenocarbonyl compounds catalyzed by bifunctional organocatalysts.

In 2011, Alemán and Marini [64] described a new strategy for the synthesis of a variety of α-alkyl, α-phenylselenyl ketones as well as their corresponding esters and amides **32**. Addition of α-selenocarbonyl derivatives **30** and catalyzed by thiourea or squaramide cinchona catalysts gave nitroalkenes **31** (Scheme 10). This reaction was carried out at low catalyst loading with excellent chemoselectivity (up to >99% *ee*, up to >98:2 dr).

**Scheme 11:**
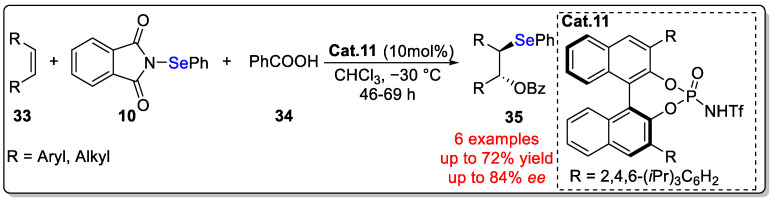
Catalytic asymmetric oxyselenenylation of olefins by chiral Brønsted acid.

In 2012, Shi and coworkers [65] used *N*-triflyl phosphoramide **Cat.11** via Brønsted acid catalysis for enantioselective oxyselenenylation of olefins (Scheme 11). Olefins **33** were enantioselectively desymmetrized with *N*-phenylselenophthalimide **10** and benzoic acid **34** in the presence of **Cat.11**, generating the chiral selenide target product **35** with moderate to good enantioselectivities (25–84% *ee*). Surprisingly, when compared to sulfenamide, the Se reagent displayed higher reactivity toward olefins.

**Scheme 12:**
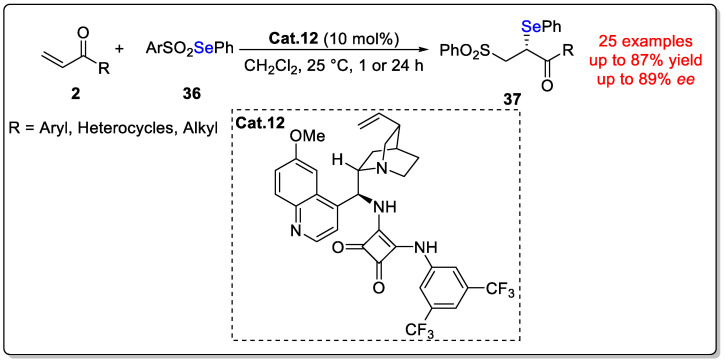
Enantioselective addition of selenosulfonates to α, β-unsaturated ketones.

In 2018, Yan and coworkers [66] reported the first organo-catalyzed enantioselective addition of selenosulfonate **36** to α, β-unsaturated ketones **2** (Scheme 12). A chiral bifunctional squaramide produced from quinine **Cat.12** are an efficient catalyst. The desired α-selenylated ketones **37** were obtained in good yield with high enantioselectivity (up to 87% yield and 89% *ee*). They can be efficiently transformed into useful building blocks with a propenylic stereocenter.

**Scheme 13:**
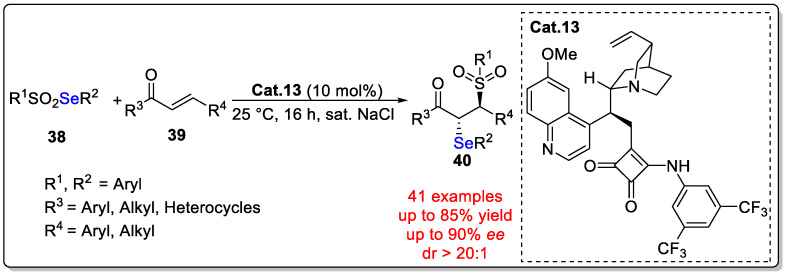
Organocatalytic enantioselective addition of selenosulfonates to α, β-unsaturated ketones.

Later, Qin and Mao [67] also reported the use of chiral squaramide **Cat.13** in the enantioselective catalysis of selenosulfonate **38** to α, β-unsaturated ketones **39** in saturated NaCl solution (Scheme 13). High yields and enantioselectivity were achieved for a series of α-selenyl and β-sulfonyl ketones, with two contiguous stereogenic centers, (up to 85% yield, 90% *ee* and dr > 20:1). Furthermore, they demonstrated effective stereocontrol in transforming chiral α-selenyl and β-sulfonyl ketones **40** into their corresponding alcohols.

**Scheme 14:**
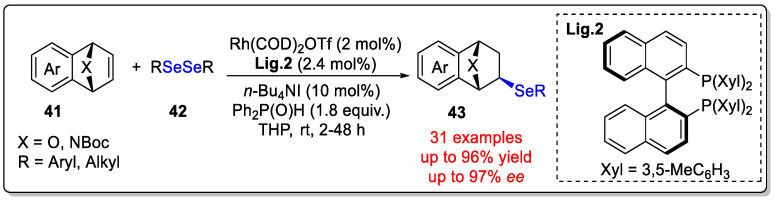
Rh-catalyzed enantioselective hydroselenation of heterobicyclic alkenes.

In 2020, Wang and Bian [68] developed the Rh(I)/(S)-Xyl-Binap catalytic system for the asymmetric hydroselenation of various nonpolar olefins 41 with diselenides 42 to produce selenol-incorporated adducts 43 (Scheme 14). By overcoming self-promoted racemic hydroselenation, a number of heterobicyclic alkenes produced selenol-incorporated adducts with high yields (up to 96%) and exceptional enantioselectivities (up to 97% ee). The approach was also used for kinetic resolution of unsymmetric oxabenzonorbornadiene.

**Scheme 15:**
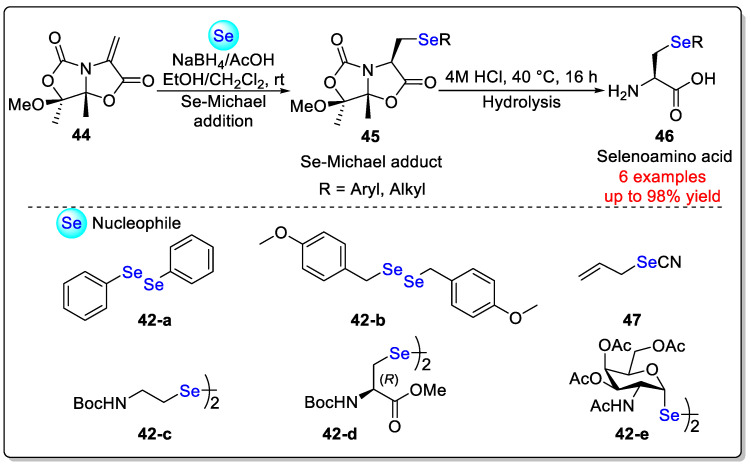
Stereoselective *Se*-Michael addition preparation of enantiomerically pure β-selenium-α-amino acids.

In 2021, Peregrina and coworkers [69] reported the first entirely chemo- and diastereoselective 1,4 conjugate additions of various Se-nucleophiles. These were generated in situ from diselenide derivatives by the action of sodium borohydride, to chiral bicyclic dehydroalanine (Dha) **44** (Scheme 15). Only single diastereomers **45** were formed in the *Se*-Michael addition reaction on Dha **44**. The results proved that the reaction was completely diastereoselectivity controlled. Acidic hydrolysis of *Se*-Michael adduct **45** resulted in the formation of enantiopure selenocysteine (Sec) derivatives **46**, that have significant potential for chemical biology applications [70].

**Scheme 16:**
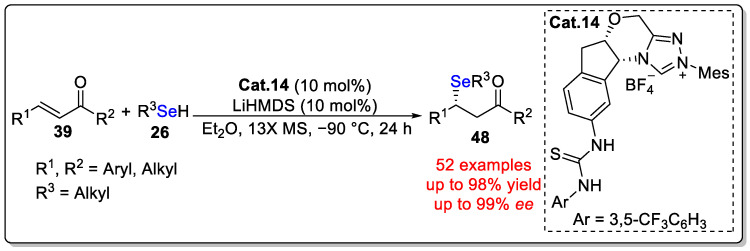
NHC catalyzed enantioselective *Se*-Michael addition reactions.

In 2022, Huang and Chen [71] reported a chiral bifunctional N-heterocyclic carbine (NHC)/thiourea catalyzed *Se*-Michael conjugate addition reaction between α, β-unsaturated ketones **39** and alkyl selenol **26** (Scheme 16). Synthesis of various chiral β-selenol carbonyl derivatives **48** were achieved with excellent yields (up to 98%) and enantioselectivities (up to 99% *ee*). The (NHC)/thiourea catalyst addresses the issue of reversibility due to the high nucleophilicity and leaving group ability of selenols, which was the main challenge in the development of asymmetric *Se*-Michael addition reactions. Both the NHC and thiourea moiety were required to achieve high enantioselectivity.

**Scheme 17:**
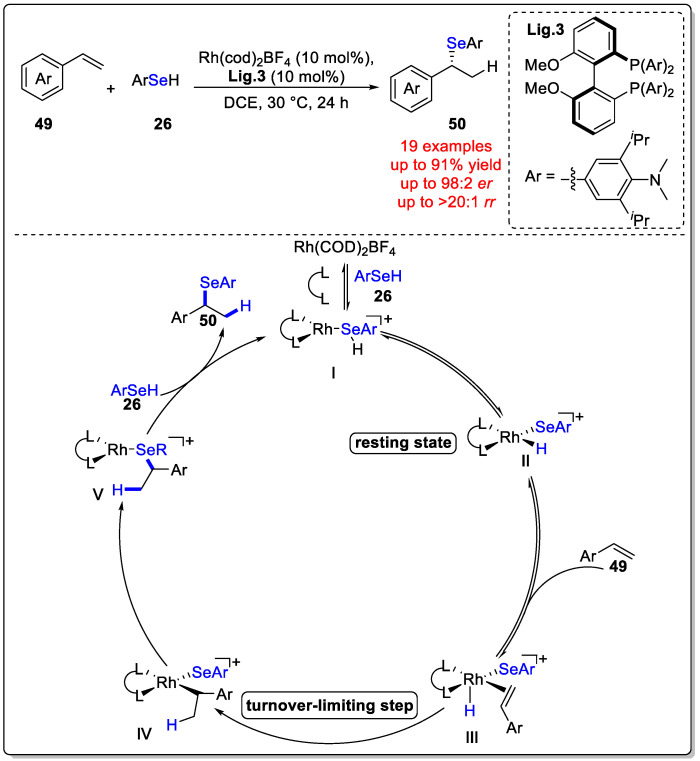
Rh-catalyzed enantioselective hydroselenation of styrene.

In 2022, Yang and Dong [72] reported the first Rh(cod)_2_BF_4_/MeO-BIPHEP **Lig.3** catalyzed enantioselective Markovnikov hydroselenation of selenols **26** with styrene **49** (Scheme 17). The desired chiral selenides were obtained in moderate to high yield (up to 91%) with good enantioselectivities (up to 98:2 er) and excellent regioselectivity (>20:1 *rr*). Based on their previous mechanistic studies, it was speculated that the reaction forms C-Se bonds with excellent regio- and enantiocontrol via a Rh-hydride pathway. Rh(I) catalysts can undergo oxidative addition to Se−H bonds to form Rh hydrides [73]. Here Rh(I) catalysts are added to ArSeH **26** to generate the Rh complex (**I)** to yield Rh−H (**II)**, a resting state of the catalytic cycle validated by NMR studies. The olefin coordinates to intermediate (**II**) and provides (**III**). Olefin migration to the Rh−H bond, providing intermediate (**IV**). Intermediate (**IV**) is reductively eliminated to yield intermediate (**V**), which is subsequently dissociated with product **50** to regenerate the Rh catalyst (**I**).

**Scheme 18:**
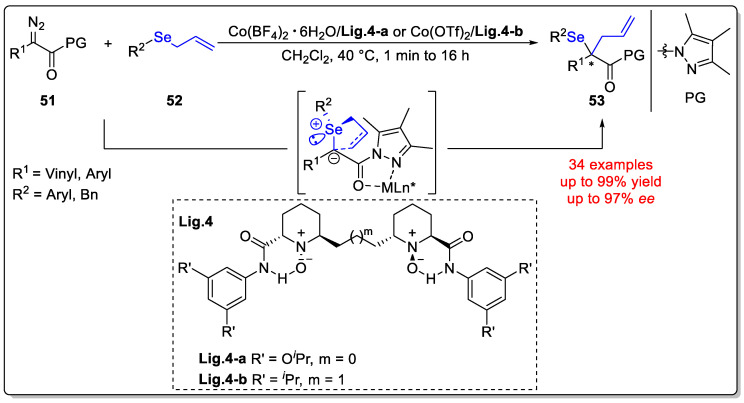
Asymmetric synthesis of selenium-containing 4H-3,1-benzoxazines.

In 2022, Feng and Liu [74] reported a novel asymmetric [2,3]-sigmatropic rearrangement of allylic selenides **52** with α-diazo pyrazoleamides **51** catalyzed by chiral *N*,*N*′-dioxide cobalt(II) complex. It is an efficient synthetic method for the preparation of optically active selenide with a quaternary C-Se stereocenter (Scheme 18). The reactions were done with 0.5–5 mol% catalyst loading and afforded chiral selenides **53** in up to 99% yield and 97% *ee*. The control experiments revealed that allyl selenide had high reactivity. Chiral N, N′-cobalt dioxide ligands and α-diazo pyrazoleamide had obvious superiority in the [2,3]-sigmatropic rearrangement reaction. The mechanistic studies have shown that the key to asymmetric rearrangement of allyl selenium ylides involves the transfer of chirality from the stable chiral selenium to the carbon of the product.

**Scheme 19:**
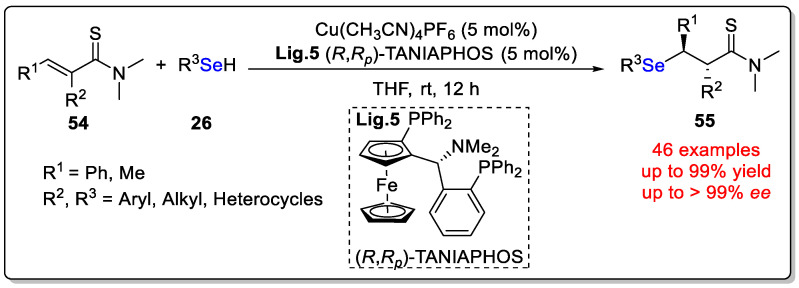
Asymmetric selenization reaction of selenol and α, β-unsaturated thioamide.

In 2023, Yin and coworkers [75] reported a Cu(CH_3_CN)_4_PF_6_/(*R*,*R_p_*)-TANIAPHOS **Lig.5** catalyzed asymmetric conjugation/protonation with α -substituted α,β-unsaturated thioamide **54** and selenols **26** (Scheme 19). More than 40 examples of α-chiral β-selenothioamide **55** were generated in high to excellent yields (up to 99%) and enantioselectivities (up to >99% *ee*). The catalytic system was also successfully applied to the asymmetric selenization of β-substituted α, β-unsaturated thioamides (2 examples) with high yields (up to 86%) and excellent enantioselectivities (up to 99% *ee*).

#### 2.1.5. Asymmetric Selenization of Aldehydes/Ketones

**Scheme 20:**
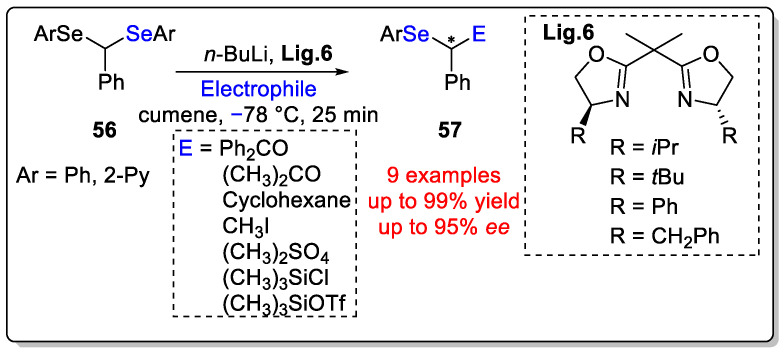
Enantioselective selenization of diselenoacetals.

In 2004, Toru and colleagues [76] reported that α-seleno carbanions derived from bis (phenylseleno) acetal **56** and bis (2-pyridylseleno) acetal **56** react enantioselectively in the presence of chiral bioxazoline **Lig.6** with electrophiles giving products **57** with excellent reactivity and high enantioselectivity (Scheme 20). The authors evaluated the reactivity of several electrophiles and found good enantioselectivity (95% *ee)*, demonstrating the method’s viability.

**Scheme 21:**
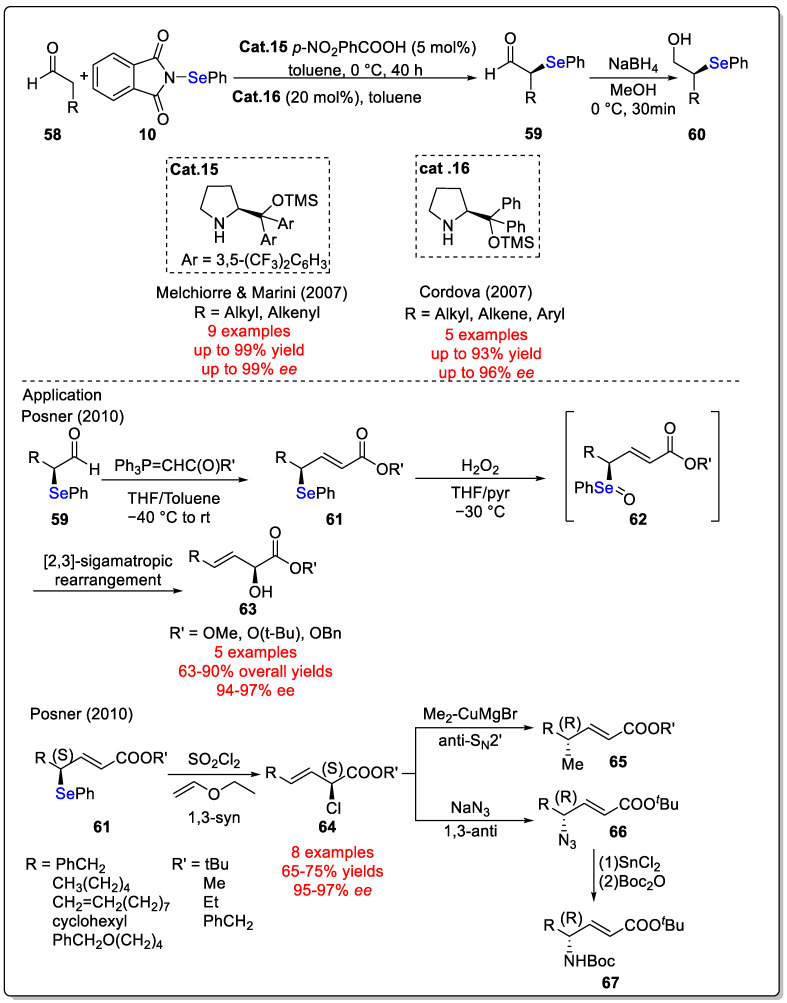
Organocatalytic asymmetric α-selenenylation of aldehydes and its application.

In 2007, Melchiorre and Marini [77] reported the application of an enamine activation strategy in the first highly enantioselective α-selenenylation of aldehydes **58** using a chiral secondary amine as catalyst and *N*-(phenylseleno)phthalimide **10** as the electrophilic selenium source (Scheme 21). Diarylprolinol silyl ethers salt (**Cat.15·**p-NO_2_PhCOOH) exhibited excellent selectivity and had higher catalytic activity. Various aldehydes, including alkyl, alkenyl, and hetero-substituted groups were tolerated in the reaction and afforded α-seleno aldehyde **59**, which was further reduced, in situ with NaBH_4_, to the corresponding chiral alcohol **60** (up to 99% yields, up to 99% *ee*) without loss of optical purity. Later, a similar approach was undertaken by Córdova and coworkers [78] who reported a highly enantioselective α-selenenylation between aldehydes **58** and *N*-(phenylseleno)phthalimide **10**. The reaction was directed using diarylprolinol silyl ethers (**Cat. 16**) as catalyst and gave the desired product α-selenoaldehydes and β-selenoalcohols in 63–93% yield with up to 96% *ee*.

In 2010, Posner and coworkers [79] reported the application of α-seleno aldehyde **59** for the synthesis of α-hydroxy-(E)-β,γ-unsaturated esters **63** in a two-step method (Scheme 21). The highly enantioselective α- seleno aldehyde **59**, synthesized in one step by asymmetric organocatalytic α-selenylation of aldehydes, was directly subjected to in situ Wittig reaction to give allylic selenide **61**. This was oxidized to selenoxide **62** using H_2_O_2_ which resulted in the spontaneous [2,3]-sigmatropic rearrangement, giving the target compound **63** in 43–65% overall yield and in 94–97% *ee*. Posner and coworkers [80] soon reported the application of α-seleno aldehyde **59** for the synthesis γ-substituted-α, β-ethylenic esters. Chiral γ-seleno-α, β-ethylenic esters **61**, were, prepared using the Wittig reaction, and treated with sulfonyl chloride and ethyl vinyl ether in hexane to give α-chloro-β, γ-ethylenic esters **64** in 65–75% yield and with 95–97% *ee*. These allyl chloride compounds react with Me_2_-CuMgBr and sodium azide to yield γ-substituted-α, β-ethylenic esters **65.** The allylic chlorides **64** were treated with sodium azide to produce the azide-substituted product 66, which was then reduced in situ using stannous chloride to the amine. This was then treated with di-tert-butyl dicarbonate affording the N-Boc-γ-amino-α, β-ethylenic tert-butyl esters **67**. Chiral racemic allyl chloride is a multifunctional, stereochemically stable electrophilic chiral compound with good chiral control and that can perform various nucleophilic substitution and olefin addition reactions.

**Scheme 22:**
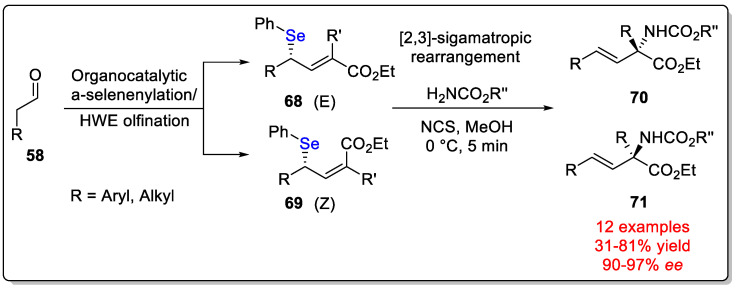
Synthesis of highly enantioselective α-alkyl, α-vinyl amino acids.

In 2011, Armstrong and his coworker [81] developed a method to synthesize chiral α-alkyl, α-vinyl amino acids **70** and **71**(quaternary vinyl glycine derivatives) using allylic selenimides as the raw material via [2,3]-sigmatropic rearrangement (Scheme 22) with excellent enantioselectivity (90–97% *ee*). The trisubstituted allylic selenides **68** and **69** were prepared by asymmetric organocatalytic α-selenenylation reaction of aldehydes **58** and Horner-Wadsworth-Emmons (HWE) olefination. Both enantiomers **70** and **71**, which were obtained after rearrangement of the (*E*)- and (*Z*)- geometric isomers **68** and **69**, exhibit excellent enantioselectivity.

**Scheme 23:**
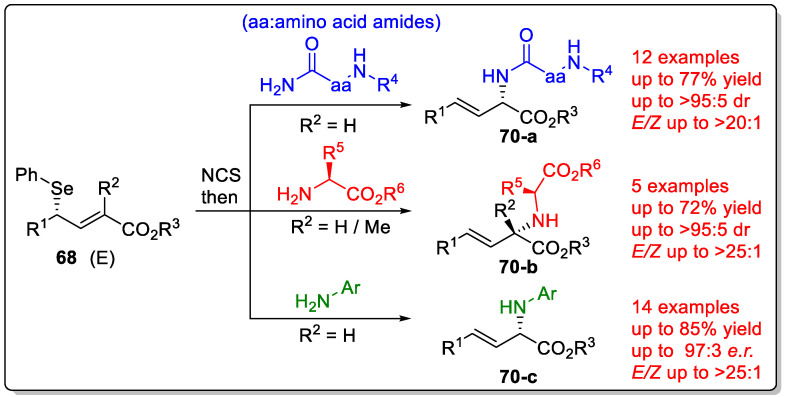
[2,3]-Sigmatropic Rearrangement of Allylic Selenimides.

In 2014, Armstrong and coworkers [82] then developed the NCS-mediated amination/[2,3]-sigmatropic rearrangement of enantioenriched allylic selenides **68**, and provided a novel strategy that uses N-protected amino acid amides to synthesize a series of peptides containing unnatural vinyl glycine amino acids resides **70-a** (Scheme 23). This strategy was applied to N-unprotected amino acid esters to generate N, N-dicarboxymethylamines **70-b**, which is a motif found in several pharmaceuticals. Furthermore, it was modified for the aromatic amines to provide a pathway to obtain a variety of high-enantioenriched N-arylamino acids **70-c**. These three product categories highlights the versatility of the amination of allyl selenide [2,3]-sigmatropic rearrangement as a method for synthesizing enantioenriched allyl amine derivatives and broadens the substrate range to previously unreported carbamates, alkyl amines, and aromatic amines.

**Scheme 24:**
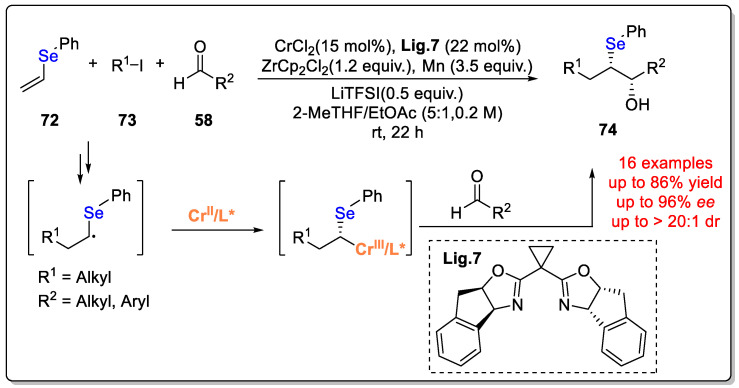
Cr-catalyzed diastereo-and enantioselective synthesis of β-hydroxy selenides.

In 2022, Wang and coworkers [83] developed a CrCl_2_/(S,R)-**Lig.7** catalyzed three-component reaction, for the preparation of valuable chiral β-hydroxy selenides **74** having adjacent stereocenters from vinyl selenide **72**, iodide **73,** aldehydes **58** (Scheme 24). A series of chiral β-hydroxy selenides **74** had good functional compatibility and were obtained in moderate to good yields (up to 86%), high diastereoselectivity (up to > 20:1 *dr*) and enantioselectivity (up to 96% *ee*). Mechanistic investigations suggest that the reaction may occur through a secondary alkyl Cr intermediate stabilized by α-selenide groups.

#### 2.1.6. Asymmetric Ring-Opening Reaction

**Scheme 25:**
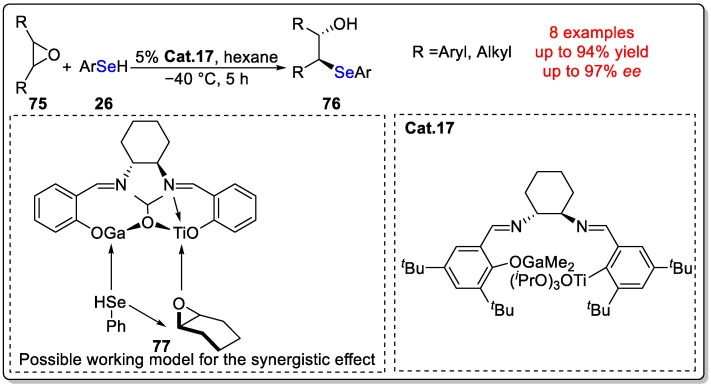
Enantioselective ring-opening reaction of epoxy compounds with aryl selenols.

In 2005, Zhu and coworkers [84] used a novel chiral Ti^IV^-Ga^III^-Salen heterometallic catalytic system **77** and obtained the optically active β-arylseleno alcohols **76** via an asymmetric ring-opening reaction with *meso*-epoxides **75** and aryl selenols **26** (up to 97% *ee*) (Scheme 25). The catalytic process revealed a strong synergistic effect of different Lewis acids in the system. The method has a good substrate scope, and a series of β-arylseleno alcohols derivatives was obtained in good to excellent enantioselectivities for both cyclic and acyclic *meso*-epoxides. However, the reaction mechanism remains unclear. Epoxides are likely to coordinate and activate the hard Lewis acid titanium, while nucleophilic reagent selenophenol coordinates with the relatively soft Lewis acid gallium, thereby effectively and selectively directing the attack of the selenophenol to the epoxide.

#### 2.1.7. Asymmetric Substitution Reaction

**Scheme 26:**
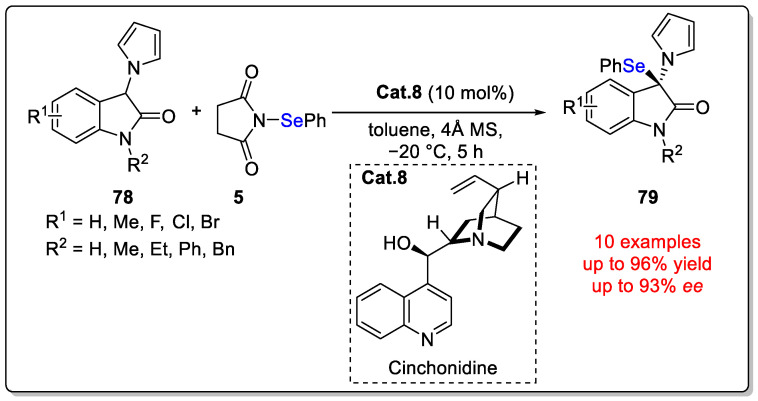
Asymmetric catalytic synthesis of 3,3-disubstituted oxyindole.

In 2015, Yuan and coworkers [85] used commercial cinchonidine **Cat.8** under mild conditions to synthesize 3,3-disubstituted oxindoles **79** via asymmetric selenenylation of 3-pyrrole-oxyindole **78** with good to excellent enantioselectivity (up to 93% *ee*) (Scheme 26). Various optically active 3-seleno-3-pyrrole-oxindoles **79** were successfully obtained. Substrate expansion experiments showed that the electronic and steric hindrance effect had little effect on the reactivity and enantioselectivity.

**Scheme 27:**
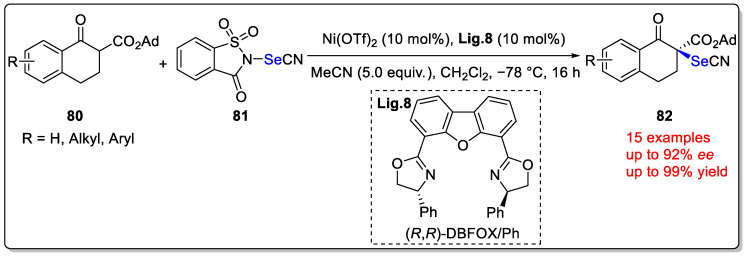
Lewis acid-catalyzed asymmetric selenocyanation of β-ketoesters with N-selenocyanatosaccharin.

In 2020, Chen and coworkers [86] reported Ni(OTf)_2_ as a Lewis acid catalyzed asymmetric selenocyanation of β-ketoesters **80** using a new selenocyanation reagent *N*-selenocyanatosaccharin **81** in the presence **Lig.8** (*R*,*R*)-DBFOX/Ph (Scheme 27). A series of α-selenocyanato-β-keto esters **82** were synthesized with good yields (up to 99%) and good *ees* values (up to 92%). This approach is straightforward, highly enantioselective, and appropriate for the synthesis of chiral selenocyanates. This is the first report on the asymmetric formation of the C(sp^3^)-SeCN bond.

**Scheme 28:**
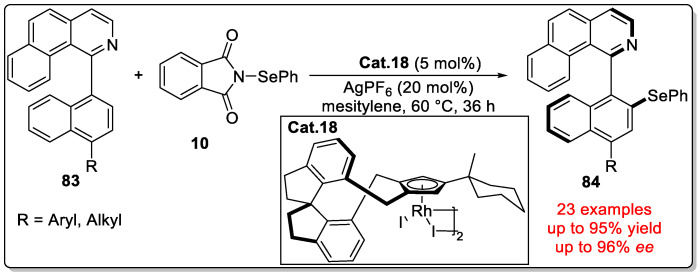
Rh(III)-catalyzed atroposelective C−H selenylation of 1-aryl isoquinolines.

In 2024, You and coworkers [87] achieved rhodium(III)-catalyzed **Cat.18** atroposelective C−H selenylation of 1-aryl isoquinolines **83** (Scheme 28). The C−H selenylation reaction between 1-aryl isoquinolines **84** and 2- (phenylselanyl)isoindoline-1,3-dione in **10** the presence of the chiral SCpRh(III) complex afforded a series of axial chiral 1-aryl isoquinoline selenides **84** in up to 95% yield and 96% *ee*. DFT calculations show that the C-Se bond formation step is via the S_N_2 pathway.

#### 2.1.8. Asymmetric Decarboxylation Reaction

**Scheme 29:**
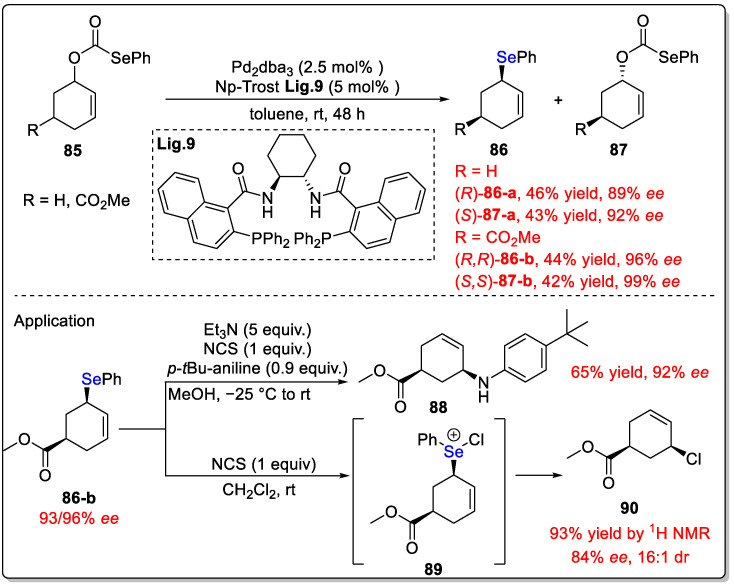
Synthesis of allyl selenides by palladium-catalyzed decarboxylative coupling.

In 2008, Tunge and coworkers [88] reported the Pd_2_dba_3_/Np-Trost **Lig.9** catalyzed decarboxylation of selenocarbonates which afforded enantioenriched allyl selenides **86** (Scheme 29) in good yields (44–46%) and excellent enantioselectivities (89–96% *ee*). The remaining unreacted selenocarbonate **87-a/b** were also isolated with excellent enantioselectivities (92–99% *ee*). The allyl selenide **86-b** underwent a [2,3]-sigmatropic rearrangement to form allylic amine **88** with high enantioselectivity (92% *ee*), or was converted to allyl chloride **90** by treatment with NCS in CH_2_Cl_2_ under mild conditions.

### 2.2. Substrate Control Enantioselectivity

**Scheme 30:**
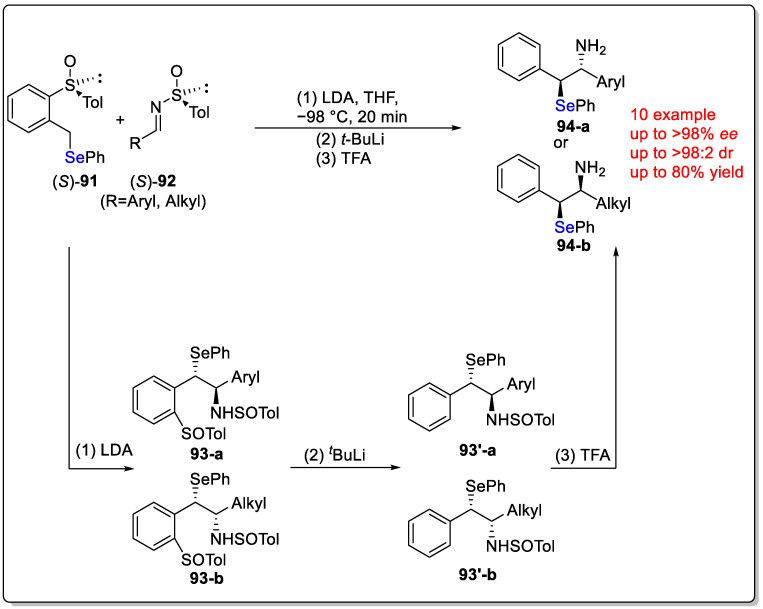
Strategy for asymmetric synthesis of enantiomerically pure 1,2-selenoamines.

In 2012, García Ruano and Degl’Innocenti [89] reported the synthesis of enantiomerically pure 1,2-selenoamines with two stereocenters using (*S*)-α-(phenylselenyl)-2-(p-tolylsulfiny) toluene **91** and (*S*)-N-(p-tolylsulfiny) imide **92** (Scheme 30). The character of the aliphatic or aromatic N-sulfinylimines **92** that are obtained is closely related to the syn or anti stereochemistry of the 1,2-Selenoamines (up to >98% *ee*, up to >98% dr, up to 80% yield). LDA was added to a THF solution of (*S*)-**91**, forming a delocalized benzylcarbanion. Then (*S*)-**92** was added to afford diastereomerically enriched **93-a** and **93-b**. To synthesize enantiomerically pure 1,2-selenoamines compounds **94-a** and **94-b** should be desulfinylated without affecting the C−Se bond nor their configurational integrity. This synthetic transformation was carried out for **94-a** and **94-b**, derived from aryl- and alkyl imines **93-a** and **93-b**, using a two-step sequence consisting of initial C-desulfinylation with *t*-BuLi and subsequent N-desulfinylation with TFA.

**Scheme 31:**
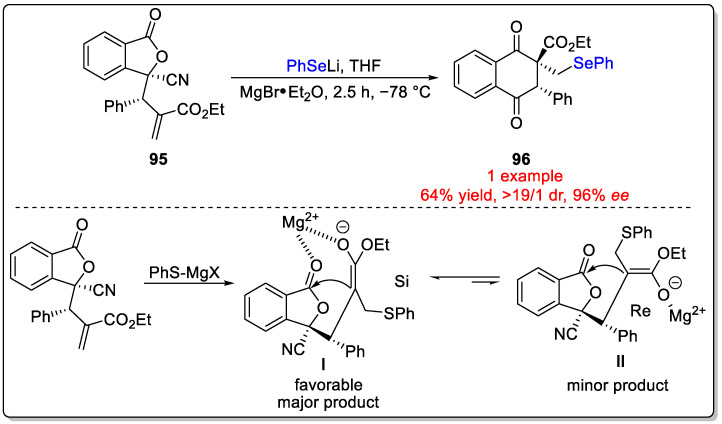
Se-Michael addition triggered ring-expansion synthesis of chiral organoselenium dihydronaphthoquinone.

In 2015, Liao and coworkers [90] reported a novel asymmetric synthesis of dihydronaphthoquinone incorporating adjacent quaternary and tertiary stereocenters via a Se-Michael addition triggered ring-expansion method (Scheme 31). Treatment of chiral phthalide **95** with lithium selenophenolate (PhSeLi), which was generated in situ from selenophenol and n-butyllithium, gave the desired chiral organoselenium **96** in good yield (64%), excellent enantioselectivity (96% *ee*) and diastereoselectivity (>19:1 dr). Although the precise mechanism by which magnesium cations control the reaction’s selectivity is not well understood, we could refer to the mechanism for the sulfonamide-Michael addition/nucleophilic addition tandem reaction [91,92,93]. The assumption is: the magnesium cation coordinates favorably to both the oxygen atom of the lactone and the enolate units of intermediate (**I**) to form a chelating structure which may contribute the current high diastereoselectivity in this tandem process.

**Scheme 32:**
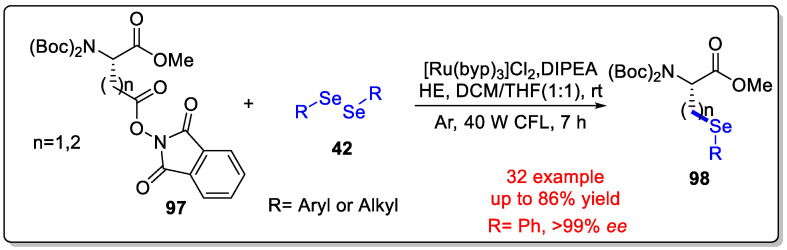
Visible-light photoredox synthesis of chiral α-selenoamino acid.

In 2016, Fu and coworkers [94] developed an efficient method for the synthesis of chiral α-selenoamino acids **98** using visible light photoredox (Scheme 32). N-Acetoxyphthalimide derivatives of two genetically coded proteinogenic amino acids, including L-aspartic acid and glutamic acid, where the carboxyls on the side chains of *N*-Bis(Boc)-Asp(OPht)-OMe (**97**, n = 1) and *N*-Bis(Boc)-Glu(OPht)-OMe (**97**, n = 2), were reacted with various diorganyl diselenides **42**. The reaction was carried out in the presence of diisopropylethylamine (DIPEA), Hantzsch ester (HE) and irradiation of 40 W compact fluorescent light (CFL) under argon atmosphere at room temperature using [Ru(bpy)_3_]Cl_2_ as the photocatalyst to afford chiral α-selenoamino acids **98**. They also proved that the visible-light photoredox decarboxylative coupling maintained the chirality of the desired products α-selenoamino acids **98** (n = 1, R = Ph, >99% *ee*).

**Scheme 33:**
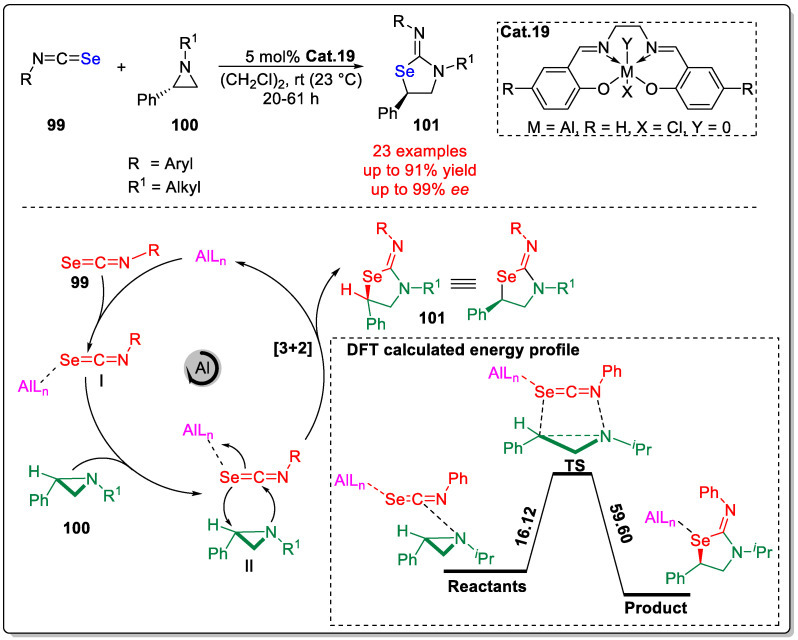
Stereospecific Al-catalysed tandem C-N/C-Se bond formation of isoselenocyanates with aziridines.

In 2018, Punniyamurthy and coworkers [95] described a new approach that involved Al-catalyzed stereospecific tandem C-N/C-Se bond formation of chiral aziridines **100** with isoselenocyanates **101** giving 90–99% *ee* and 77–91% yields (Scheme 33). The energy profile diagram for the reaction (Scheme 33, bottom right) clearly shows that it is a one-step S_N_2-type reaction. The observed experimental and DFT studies indicate that chelation of isoselenocyanate **99** with Al-salen may result in the formation of an Al-complex (**I**), which can couple with aziridine **100** via a concerted S_N_2 pathway b to produce the target products **101**.

**Scheme 34:**
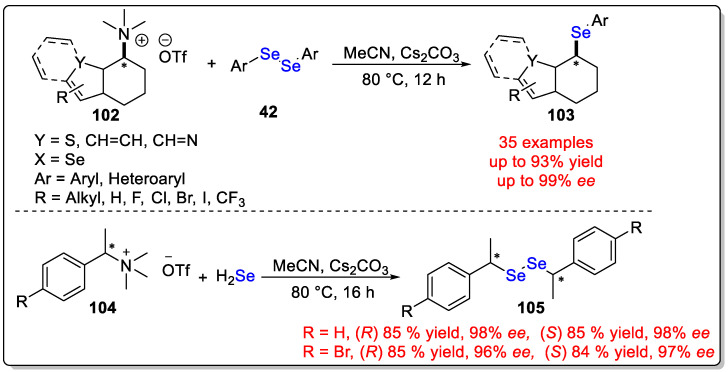
Synthesis of di(hetero)aryl diselenides and dibenzylic diselenides from aryl quaternary ammonium salts and selenium compounds.

In 2020, Zeng and coworkers [96] developed a new method to synthesize (enantioenriched) selenoethers **103** from (chiral) benzylic trimethylammonium salts **102** and di(hetero)aryl diselenides **42** (Scheme 34). Benzyl selenyl ether was synthesized under weak basic conditions without transition metal catalysis with good to excellent yields (up to 93%) and high enantiomeric purity (up to 99% *ee*). Later in 2021 they developed a novel approach for the synthesis of (enantio-enriched) dibenzyl diselenides **105** via S_N_2 nucleophilic substitution of (enantiomerically enriched) benzyl quaternary ammonium salt **104** and diselenide dianion (Se^2−^) [97].

## 3. Application of Chiral Organoselenium Compounds in the Asymmetric Catalysis

**Scheme 35:**
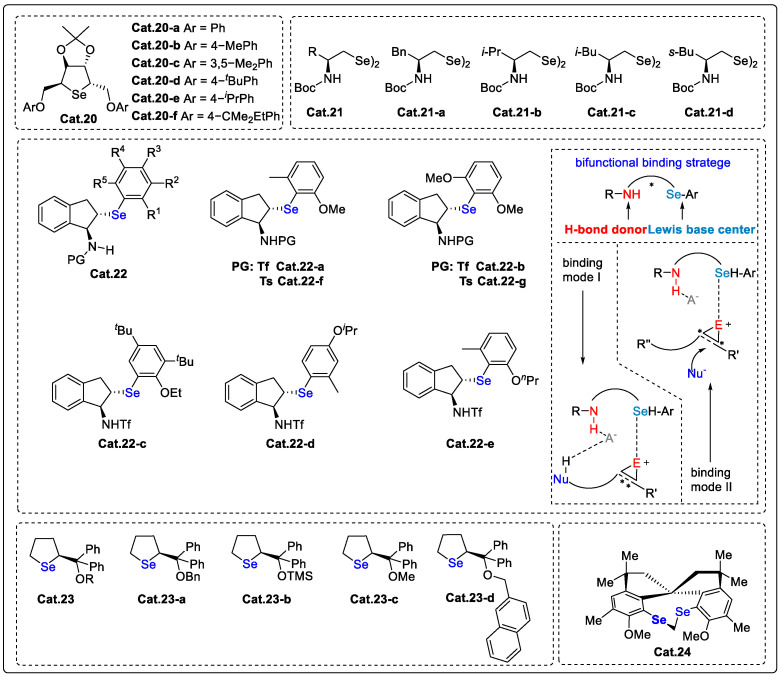
Chiral selenide catalysts.

Organoselenium compounds exhibit a wide range of chemical properties. Their use in catalysis is very relevant and well documented. Furthermore, a number of synthetic approaches to access diverse chiral organoselenium are reported. A number of chiral selenide compounds were produced and employed as catalysts in a variety of asymmetric reactions (Scheme 35).

**Scheme 36:**
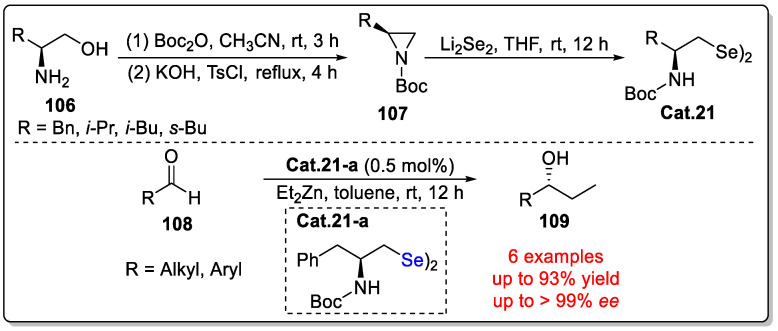
Chiral aminodiselenide compounds catalyze the synthesis of enantioselective secondary alcohols.

In 2003, Braga and coworkers [98] synthesized a series of chiral aliphatic aminodiselenide **Cat.21** by the ring-opening reaction of azidine **107** which was then used as a catalyst for the enantioselective addition of diethylzinc to aldehydes (Scheme 36). The chiral secondary alcohol derivatives **109** were produced in good to excellent yields (up to 93%) and enantioselectivities (up to >99% *ee*). In substrate expansion experiments, the effect of alkyl substituents and aryl substituted aldehydes on stereoselectivity was examined, and the results showed moderate to excellent enantioselectivities (45 - > 99% *ee*). Notably, alkyl chains have a greater effect on enantioselectivity, resulting in a significant decrease in enantioselectivity from hexanal (>99% *ee*) to decanal (45% *ee*).

**Scheme 37:**
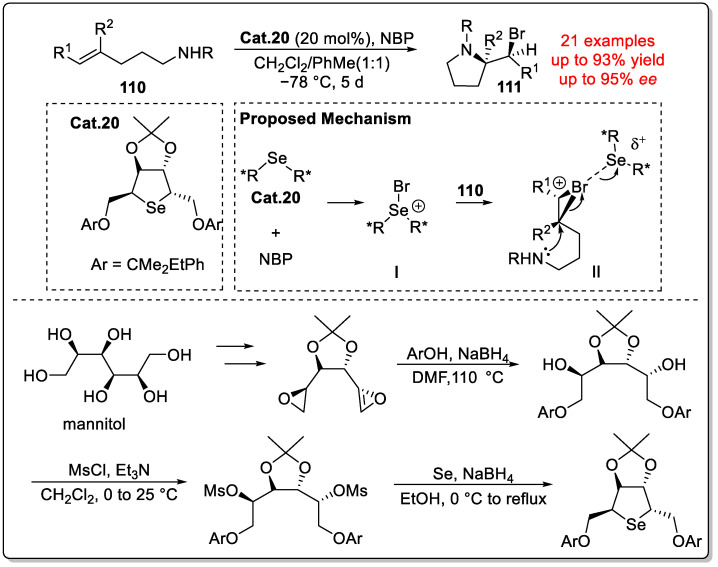
C_2_-symmetric cyclic selenium-catalyzed enantioselective bromoaminocyclization.

In 2013, Yeung and coworkers [39] used a C_2_-symmetric mannitol-derived cyclic selenium to catalyze asymmetric bromocyclization of trisubstituted olefinic amides **110** (Scheme 37, up to 95% *ee*). The resulting enantioenriched pyrrolidine derivatives, with two stereogenic centers, can undergo rearrangement to yield 2,3-disubstituted piperidines **111** with good diastereoselectivity and enantiospecificity. It is hypothesized that the mechanism of this cyclic-selenium-catalyzed bromocyclization reaction (Scheme 37) is as follows: coordination of Lewis basic selenium **Cat. 20** to NBP forms activated electrophilic brominating species (**I**). Subsequent interaction between (**I**) and the olefinic substrate **110** would give selenium coordinated bromonium intermediate species (**II**).

**Scheme 38:**
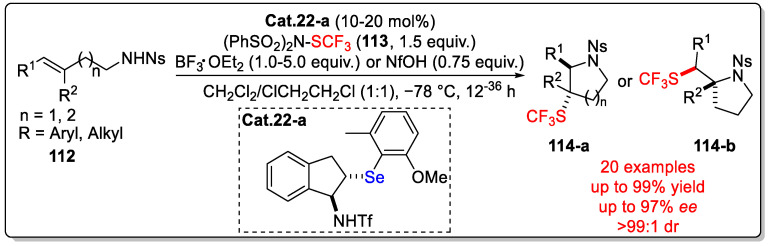
Enantioselective trifluoromethylthiolating aminocyclization.

In 2017, Zhao and coworkers [99] developed a new bifunctional chiral selenide **Cat.22-a** for catalyzing the enantioselective CF_3_S aminocyclization of olefins **112** to construct a series of saturated azaheterocycles **114-a/b** in good yields (up to 99%) high diastereo-and enantioselectivities (Scheme 38, up to 97% *ee*, >99:1 dr). This method provides a new approach for the synthesis of chiral saturated pyrrolidines and piperidines. The indane-based chiral amino aryl chalcogenide catalysts can give acceptable H-bonding by varying the amino protecting groups. They can also produce good Lewis basicity and steric hindrance by modifying the substituents on the aryl chalcogenide motifs. These features allow them to produce a chiral environment while also meeting the reactivity requirements. Notably, they have been effectively applied to several asymmetric electrophilic reactions involving alkenes, alkynes, and arenes, thus broadening the field of electrophilic reactions [100].

**Scheme 39:**
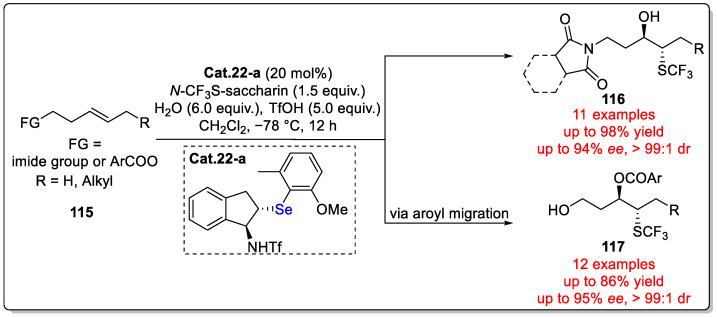
Enantioselective oxytrifluoromethylthiolation of aliphatic internal alkenes.

In 2018, Zhao and coworkers [101] again reported the use of chiral selenide **Cat.22-a** for oxytrifluoromethylthiolation of aliphatic internal alkenes **115**, for the synthesis of CF_3_S-substituted 1,3-amino alcohol **116** (up to 98% yield and up to 94% *ee*) and 1,3-diol derivatives **117** (86% yield, and 95% *ee*) with high regio-, enantio-, and diastereoselective (Scheme 39, up to >99:1 dr). In this approach, the alkenes were functionalized regioselective, with the surrounding imide or ester group providing a functional group.

**Scheme 40:**
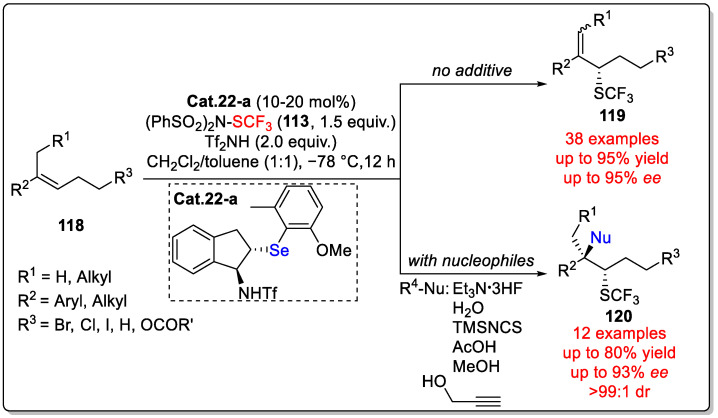
Allyl enantioselective reaction and intermolecular difunctionalization of olefins.

In another investigation in 2018, Zhao and coworkers [102] reported the use of chiral selenide **Cat.22-a** for allylic trifluoromethylthiolation of alkenes **118**, affording the chiral allylic CF_3_S-substituted products **119** in good to excellent yields (up to 95%) and excellent enantioselectivities (Scheme 40, up to 95% *ee*). The unactivated alkenes **118** also achieved intermolecular difunctionalization in the presence of a nucleophile reagent, generating the corresponding difunctionalized chiral CF_3_S-substituted products **120** in moderate to good yields (up to 80%) with excellent enantio- and diastereoselectivities (up to 93% *ee*, >99:1 dr). This method provided a new approach for the synthesis of C-SCF_3_ stereogenic compounds, where alkenes do not require directing group assistance for enantiocontrol.

**Scheme 41:**
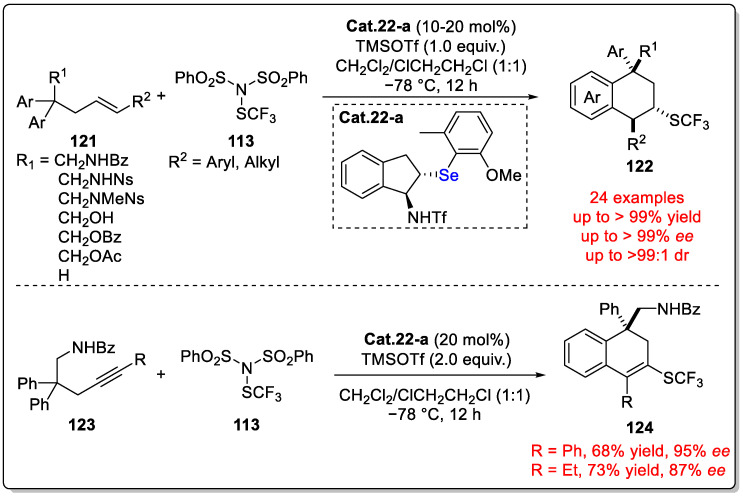
Enantioselective desymmetrization and trifluoromethylthiolation of *gem*-diaryl tethered alkenes/alkynes.

In the same year, Zhao and coworkers [103] reported the use of efficient chiral bifunctional selenide **Cat.22-a** for enantioselective desymmetrization and trifluoromethylthiolation of *gem*-diaryl tethered alkenes **121**, which gave the corresponding CF_3_S-tetrahydronaphthalene derivatives **122** in good to excellent yields (up to >99%) with excellent enantio- and diastereoselectivities (Scheme 41, up to >99% *ee*, >99:1 dr). Remarkably, diphenyl-tethered alkynes **123** also worked well in the reaction and gave the desired chiral dihydronaphthalenes compounds **124** in good yields (68% and 73%) with excellent enantioselectivities (up to 95% *ee*).

**Scheme 42:**
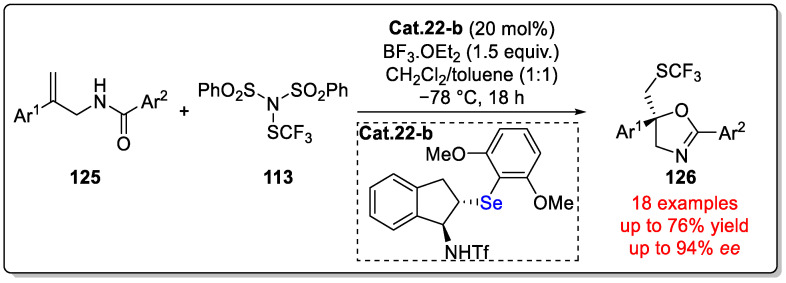
Chiral selenide-catalyzed enantioselective synthesis of trifluoromethylthiolated 2,5-disubstituted oxazolines.

In 2019, Zhao and coworkers [104] discovered a new application for chiral bifunctional selenide **Cat.22-b,** which was used for enantioselective trifluoromethyl thiolation of 1,1-disubstituted olefins **125** (Scheme 42). Various chiral trifluoromethylthiolated 2,5-disubstituted oxazoles **126** were synthesized in good yields (up to 76% yield) with good to excellent enantioselectivities (up to 94% *ee*), providing a new approach for the synthesis of chiral oxazolines.

**Scheme 43:**
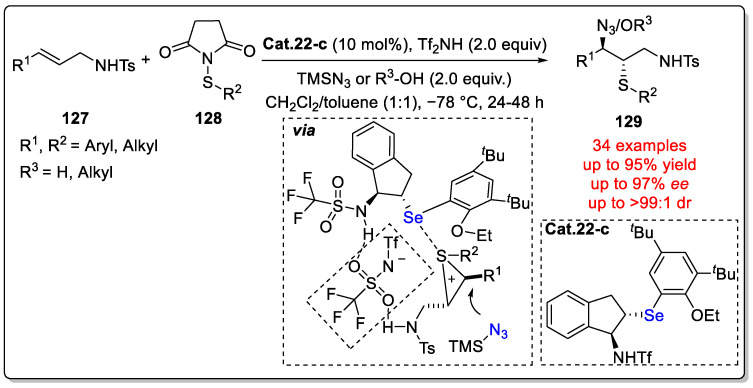
Enantioselective construction of chiral sulfides via chiral selenide-catalytic electrophilic azidothiolation and oxythiolation of *N*-allyl sulfonamides.

In 2019, Zhao and his coworker [105] reported the use of new chiral bifunctional selenide **Cat.22-c** for electrophilic azidothiolation and oxythiolation of *N*-allyl sulfonamides **127**, to construct chiral vicinal azidosulfides and oxysulfides **129** in good yields (up to 95%) with excellent enantio- and diastereoselectivities (Scheme 43, up to 97% *ee*, up to >99:1 dr). This is the first example of chiral bifunctional selenide-catalyzed enantioselective electrophilic azidothiolation and long-chain-alkylthiolation of alkenes, which indicates the possible application of this method to other alkenes. The key arylthiiranium ion intermediate in the reaction is depicted as an anion bridge Tf_2_NH which binds to the substrate and the catalyst (Scheme 42).

**Scheme 44:**
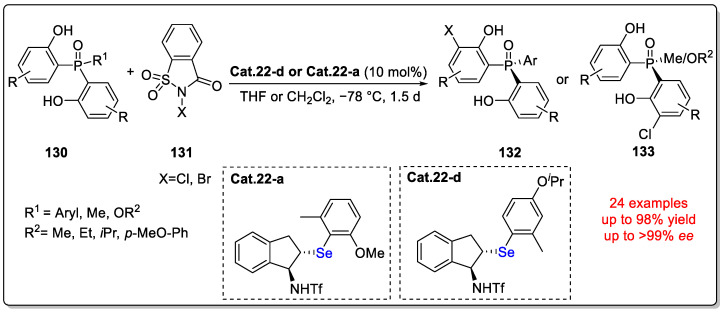
Enantioselective construction of P-chirogenic compounds via electrophilic aromatic chlorination.

In 2020, Zhao and coworkers [106] reported the use of chiral bifunctional selenide **Cat.22-d** or **Cat.22-a** for desymmetrizing enantioselective electrophilic aromatic halogenation of **130** (Scheme 44), to construct P-chirogenic triaryl phosphine oxide compounds **132** or **133** in high yields (up to 98%) with excellent enantioselectivities (up to >99% *ee*). Interestingly, chlorination switches from *ortho* to *para* positions, when the substrates were changed from triaryl phosphine oxides to alkyl diaryl phosphine oxides and diaryl phosphinates, depending on the structure of the phenols. Control experiments were carried out and the results showed that H-binding could strengthen the interaction between the catalyst and substrate to control the reactivities and racemization.

**Scheme 45:**
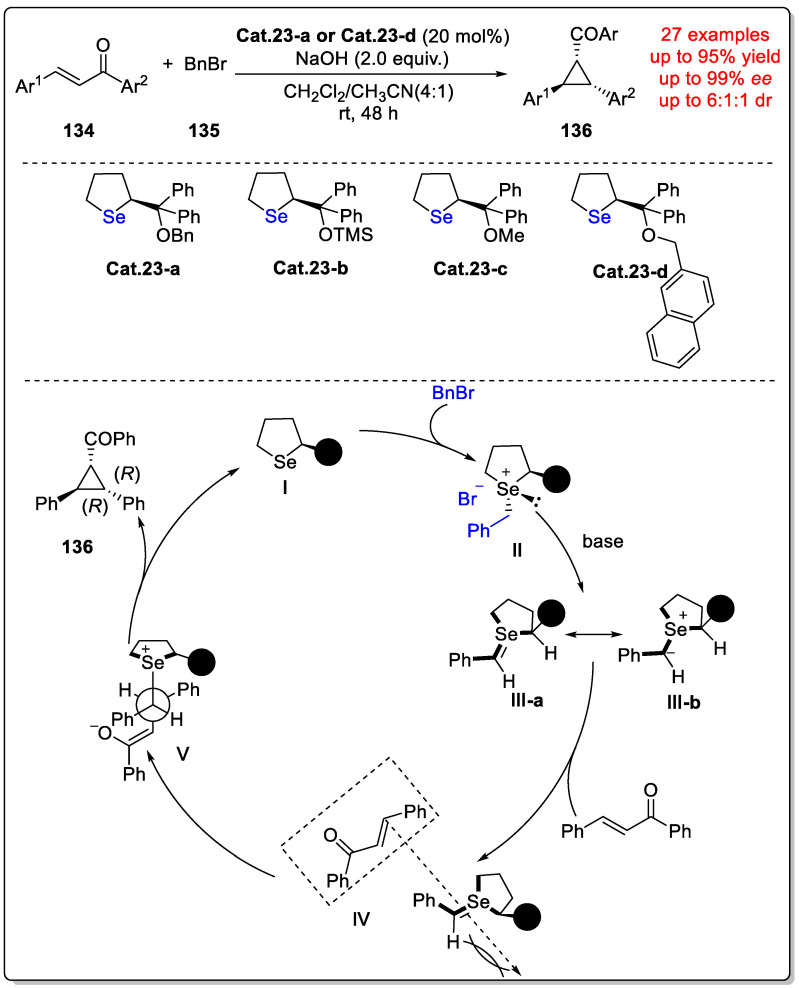
Organoselenium-catalyzed asymmetric cyclopropanations of (*E*)-Chalcones.

In 2021,Chein and coworkers [107] reported the use of a new type of chiral tetrahydroselenophene **Cat.23-a** or **Cat.23-d** (Scheme 45), which were synthesized from (*R*)-3-(3-bromopropyl)-2,2-diphenyloxirane, for asymmetric cyclopropanations of (*E*)-chalcones **134**, giving the cyclopropanes **136** with moderate to excellent yields (up to 95%) and excellent enantioselectivities (up to 99% *ee*). This is the first example of chiral selenide-catalyzed enantioselective cyclopropanation reaction. The proposed catalytic cycle (Scheme 45) shows selenide catalyst **I** is initially converted into selenonium salt **II**, and then transformed into the corresponding ylide **III-b** under basic conditions. The ylide carbanion’s unshared electrons are delocalized in the selenium atom’s 4d orbitals, forming the benzene system. Thus, the resonance structure of betaine adopts a conformation [108,109,110,111], in which the lone electron pair on the selenium remains orthogonal to the π-system of ylene **III-a**, while the phenyl group occupies a sterically relaxed pseudoequatorial position. The bulky side chain in **III** is positioned so that it blocks the *si* face of the ylide carbon, forcing the Michael receptor to be approached from the *re* face resulting in intermediate (**V**) with a preferred *anti*-*anti*-conformation.

**Scheme 46:**
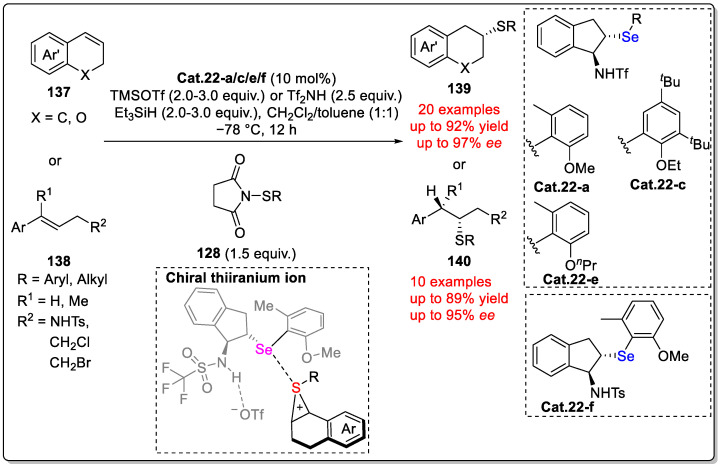
Chiral selenide-catalyzed enantioselective electrophilic hydrothiolation of Alkenes.

In 2022, Zhao and coworkers [112] reported the use of chiral bifunctional selenide, including **Cat.22-a**, **Cat.22-c**, **Cat.22-e**, and **Cat.22-f**, for enantioselective electrophilic hydrothiolation of alkenes, which was a new approach for constructing chiral sulfides (Scheme 46). In the presence of electrophilic sulfur reagents **128** and silanes, the cyclic and acyclic unactivated alkenes **137** or **138** efficiently provided various chiral sulfide products **139** or **140** with good to excellent yields (up to 92% and 89%) and excellent enantioselectivities (up to 97% *ee* and 95% *ee*). Mechanistic studies indicated a relatively stable chiral thiiranium ion is considered the key reactive intermediate.

**Scheme 47:**
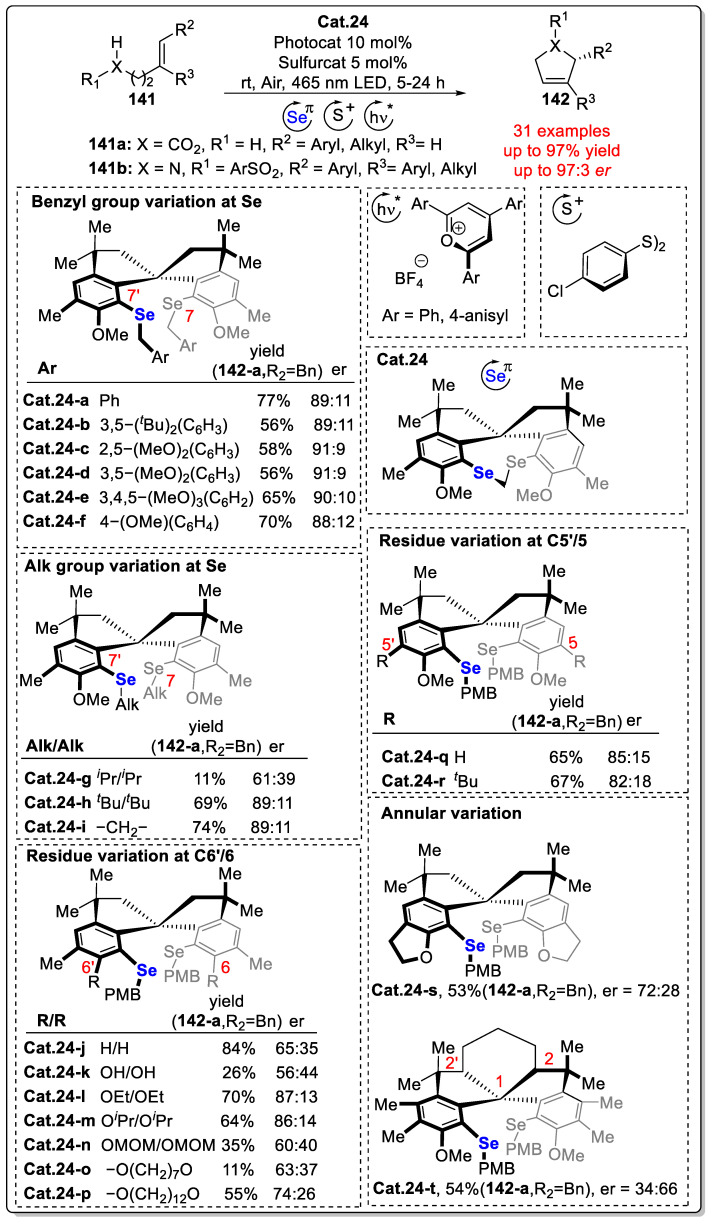
Asymmetric photoaerobic lactonization and Aza-Wacker cyclization of alkenes enabled by ternary selenium-sulfur multicatalysis.

In 2023, Breder and coworkers [41] developed a new catalyst, which was chiral and nonracemic spirobiindane selenium-π-acid catalysts **Cat.24**, for asymmetric, photoaerobic lactonization and cycloamination of non-directing alkenes **141** (Scheme 47). 2,4,6-Tris(p-anisyl)pyrylium tetrafluoroborate was worked as photoredox catalyst and 4,4′-Dichlorodiphenyldisulfide was worked as cocatalyst. Enoic acids **141a** or unsaturated sulfonamides **141b** were exposed to photoredox catalyst in combination with Se-π-acid catalysts **Cat.24** and sulfur cocatalyst under air and 465 nm irradiation for 5–24 h. Butenolides **142a** and 3-pyrrolines **142b** were obtained in good to excellent yields (up to 97%) and high enantioselectivities (up to 97:3 *er*). The authors investigated the impact of structural modifications on the catalysts **24**. The electronic effect of the benzylic Se-protecting groups (7/7′position, Scheme 46) did not show a specific trend with regards to the er values (**Cat.24 a**-**f**). However, changing the Se-protecting group from benzylic to aliphatic protecting group had a significant effect (**Cat.24 g**-**i**). Modifications at position 6/6′ indicated that the oxygen atoms may play an important role in stereoinduction (**Cat.24 j**-**p**). The results were consistent with that reported by Wirth and Tomoda et al. They demonstrated that nonbonding interactions between n → σ* O···Se typically lead in rigid catalyst−substrate conformations, resulting in higher levels of stereoinduction [113,114]. Continuing with modification at the distal 5/5′ position had no significant effect on the reaction outcome (**Cat.24 q**-**r**). Making the catalyst skeleton more rigid by adding substituents (1s/t) led to weaker stereoinduction (**Cat.24 s**-**t**). For **Cat.24-s**, the author interpreted this as a result of conformational restrictions at the 6/6′O atoms, which potentially prevents n → σ* O···Se overlap.

**Scheme 48:**
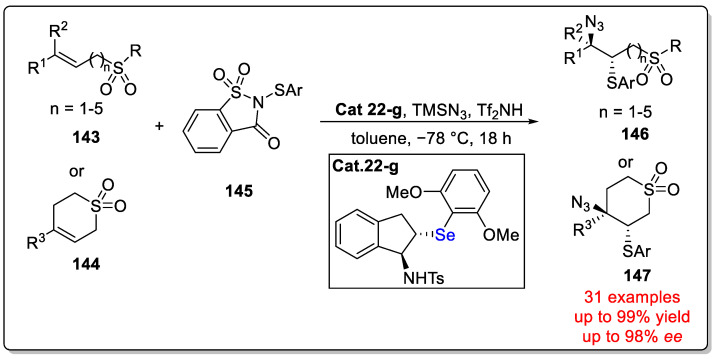
Catalytic enantioselective electrophilic difunctionalization of unsaturated sulfones.

In 2024, Zhao and coworkers [115] reported an efficient strategy of enantioselective thiolative azidation of sulfone-tethered alkenes **143** or **144** via a chiral chalcogenide **Cat.22-g** catalyzed electrophilic reaction (Scheme 48). A series of enantioenriched sulfones **146** or **147** bearing remote stereogenic centers was synthesized with good to excellent yields (up to 99%) and high enantioselectivities (up to 98% *ee*) with linear unsaturated sulfones and cyclic unsaturated sulfones.

## 4. Conclusions

In conclusion, the synthesis of chiral organoselenium compounds and their application as a catalyst in asymmetric synthesis over the past decade was summarized in this review. The common approach is to use chiral catalysts or chiral ligands to regulate enantioselectivity for asymmetric synthesis. Another method is to use the chiral structure of the raw material to control the stereoselectivity of the reaction process. In recent years, chiral organoselenium compounds have been developed as catalysts in asymmetric synthesis, facilitating the synthesis of a variety of target products with high enantioselectivity.

Although the synthesis of chiral organoselenium compounds has achieved significant progress, there are limited reactions for the use of chiral catalysts or chiral ligands. The synthetic route for these reactions is complex or not commercialized, which represents a further limitation of this method. Currently, controlling enantioselectivity through chiral catalysts or chiral ligands is still the principal method in asymmetric synthesis. Therefore, the development of a green and economical method [75] for the preparation and synthesis of chiral selenium compounds has a bright prospect.

## Data Availability

No new data were created or analyzed in this study. Data sharing is not applicable to this article.
